# A Veritable Menagerie of Heritable Bacteria from Ants, Butterflies, and Beyond: Broad Molecular Surveys and a Systematic Review

**DOI:** 10.1371/journal.pone.0051027

**Published:** 2012-12-20

**Authors:** Jacob A. Russell, Colin F. Funaro, Ysabel M. Giraldo, Benjamin Goldman-Huertas, David Suh, Daniel J. C. Kronauer, Corrie S. Moreau, Naomi E. Pierce

**Affiliations:** 1 Department of Biology, Drexel University, Philadelphia, Pennsylvania, United States of America; 2 Department of Organismic and Evolutionary Biology, Harvard University, Cambridge, Massachusetts, United States of America; 3 Field Museum of Natural History, Department of Zoology, Chicago, Illinois, United States of America; University Of Montana – Missoula, United States of America

## Abstract

Maternally transmitted bacteria have been important players in the evolution of insects and other arthropods, affecting their nutrition, defense, development, and reproduction. *Wolbachia* are the best studied among these and typically the most prevalent. While several other bacteria have independently evolved a heritable lifestyle, less is known about their host ranges. Moreover, most groups of insects have not had their heritable microflora systematically surveyed across a broad range of their taxonomic diversity. To help remedy these shortcomings we used diagnostic PCR to screen for five groups of heritable symbionts—*Arsenophonus* spp., *Cardinium hertigii*, *Hamiltonella defensa*, *Spiroplasma* spp., and *Wolbachia* spp.—across the ants and lepidopterans (focusing, in the latter case, on two butterfly families—the Lycaenidae and Nymphalidae). We did not detect *Cardinium* or *Hamiltonella* in any host. *Wolbachia* were the most widespread, while *Spiroplasma* (ants and lepidopterans) and *Arsenophonus* (ants only) were present at low levels. Co-infections with different *Wolbachia* strains appeared especially common in ants and less so in lepidopterans. While no additional facultative heritable symbionts were found among ants using universal bacterial primers, microbes related to heritable enteric bacteria were detected in several hosts. In summary, our findings show that *Wolbachia* are the dominant heritable symbionts of ants and at least some lepidopterans. However, a systematic review of symbiont frequencies across host taxa revealed that this is not always the case across other arthropods. Furthermore, comparisons of symbiont frequencies revealed that the prevalence of *Wolbachia* and other heritable symbionts varies substantially across lower-level arthropod taxa. We discuss the correlates, potential causes, and implications of these patterns, providing hypotheses on host attributes that may shape the distributions of these influential bacteria.

## Introduction

Insects colonize nearly every terrestrial habitat on the planet, having diversified into millions of extant species. Their roles as pollinators, herbivores, predators, parasites, and mutualists make them integral parts of terrestrial ecosystems, and their biomass within these habitats is largely unrivaled by other animals. Across these invertebrates, the evolutionary innovations enabling adaptation, niche shifts, and diversification have primarily been driven by mutations in their endogenous genomes. Yet, exogenous agents have also played roles in these processes, as many insects harbor maternally transmitted bacteria that provide an additional source of genetic variation with adaptive potential [1].

The variety of these heritable symbionts is impressive, with bacteria from multiple families, orders, and phyla having evolved this highly specialized lifestyle [1]. Several have also independently evolved similar strategies to spread within host populations, making their living through manipulation of host reproduction, or through benefits to host nutrition, defense, or environmental tolerance [2]–[7]. These effects have enabled heritable symbionts to shape the ecology and evolution of their hosts, and occasional instances of horizontal transfer between species [8]–[12] have allowed their impacts to be disseminated across the insects and beyond.

*Wolbachia* are by far the best known of the maternally transmitted symbionts. These intracellular members of the Alphaproteobacteria infect a majority of the world's insect species, and they are also found in isopods, arachnids, and nematodes [13]–[16]. Other heritable bacteria are typically less prevalent across the arthropods, with lower frequencies and seemingly patchier distributions across many host groups [17]–[21]. Since their overall prevalence can vary among host taxa, it is likely that some possess host range restrictions or that some insect groups are either especially accommodating or especially inhospitable. Patchy distributions are also apparent, to some extent, for *Wolbachia*. Although few host groups appear to be off-limits to these microbes, several are rarely infected, just as some host taxa show impressively high rates of *Wolbachia* infection [22]–[24]. In addition, some *Wolbachia* lineages are primarily confined to a limited range of related arthropods [25], [26], suggesting recently derived host range restrictions.

Despite our expanding knowledge of symbiont distributions, outside of the genus *Drosophila* [27] most insects have not been screened with great depth or breadth at lower taxonomic levels. When we consider that *Wolbachia* frequencies can differ widely across related host families, and even related genera [28], it becomes apparent that this lack of information has hindered our understanding of the factors that shape symbiont distributions. Given the known effects of heritable bacteria, surveys for such microbes are likely to identify bacterial species with profound impacts on the nutritional ecology, defensive interactions, reproduction, development, and genome evolution of their host arthropods [2], [29].

To further elucidate their distributions, we utilized a series of molecular approaches to study five heritable symbionts across the moths, butterflies (Insecta: Lepidoptera) and ants (Insecta: Hymenoptera: Formicidae). Targeted bacteria included 1) *Arsenophonus* spp., 2) *Cardinium hertigii*, and 3) *Spiroplasma* spp. found previously across 4–7% of insect and arachnid species [17]; 4) *Hamiltonella defensa*, a heritable symbiont primarily known from whiteflies and approximately 10–15% of surveyed aphid species [4], [30], [31]; and 5) *Wolbachia* spp., found typically at levels ranging from 15–35% in most diagnostic screening studies [16]. While these bacteria differ in their range of phenotypic effects, all are typically non-essential associates, infecting less than 100% of the individuals within infected species. Additionally, all five symbionts execute at least one type of reproductive manipulation in some host backgrounds, and all but *Cardinium* have been implicated as defensive mutualists [32]–[36]. With the exception of *Hamiltonella*, all symbionts have been previously targeted in systematic screening studies, including a recent study across a wide variety of arthropods [17]. These prior efforts provide a wealth of data on symbiont frequencies for comparison with our results. *Hamiltonella*, in contrast, was targeted due to its presence in some sap-feeding insects that are symbiotic with ants, suggesting the possibility for horizontal transfer.

For a subset of the targeted ants, we also searched for heritable symbionts through surveys of 16S rRNA libraries amplified with universal primers, focusing on species known to harbor maternally transmitted symbionts (from the tribe Camponotini) and those screening ambiguously with diagnostic PCR. Our molecular screening and sequence analyses allowed us to estimate symbiont incidence across species, prevalence within host taxa, and both diversity and dominance of bacteria within single individuals. To place our results into a broader context, we also conducted a systematic review of symbiont frequencies across arthropod taxa, compiling results from 115 prior studies that used diagnostic PCR to screen for heritable bacteria. Combined, our efforts have yielded one of the most comprehensive examinations of symbiont distributions to date.

## Methods

### Insect specimens

In total, our surveys targeted 987 specimens of ants, moths, and butterflies. A total of ∼259 lepidopteran species (approximation due to the presence of unidentified species from the same genera and, thus, possible redundancy) from 13 families, 37 subfamilies, and 195 genera were surveyed for one or more of the targeted bacteria using diagnostic PCR. The majority of surveyed Lepidoptera hailed from the butterfly families Lycaenidae and Nymphalidae. Sampling within the Lycaenidae targeted 93 genera across 30 of 33 tribes, and six of seven subfamilies. Within the Nymphalidae we screened across 77 genera from 9 out of 12 subfamilies and 35 of 40 tribes. Ants targeted in diagnostic PCR screening came from ∼406 ant species spanning 19 of 21 known subfamilies, and 124 genera. A large percentage of the targeted ants were those used to construct the first major ant phylogeny [37], comprising a good representation of the diversity across this family. In addition, high representation within the genus *Pheidole*, the army ants, and the genus *Polyrhachis* (from the Australian Wet Tropics) enabled us to screen extensively within several focal groups of ants. This allowed us to estimate intraspecific infection frequencies for some species, while also enabling infection frequency comparisons among related groups of ants. Surveys in the genus *Polyrhachis* were of special interest due to the group's known associations with heritable *Blochmannia* symbionts and the possible presence of facultative, “secondary” symbionts.

Collections were made previously from a number of temperate and tropical locations across all continents except Antarctica. All necessary permits were obtained for the described field studies. Army ant samples from Uganda were collected under a permit from the Uganda National Council for Science and Technology (EC483), while from Kenya were collected under permits from the National Museums of Kenya (NMK/CBD/10/VOL.2) and the Kenya Ministry of Education, Science and Technology (MOEST13/001/31C168/5 and MOEST13/001/31C168/2). Additional collection information can be found within previously published papers on the specimens used in this study [26], [37], [38].

### General molecular methodologies

Information on DNA extractions, PCR conditions, primer design, electrophoresis, positive and negative controls, template quality assays, PCR product purification, cloning, and sequencing can be found in Information S1 and Table S1. Specific details on the primers utilized for diagnostic screening, universal PCR, and sequencing can be found in Table S2.

### General phylogenetic methodologies

Sequences generated with universal or diagnostic PCR primers (along with their closest relatives) were aligned on the RDP website [39]. Maximum likelihood phylogenies were constructed on the CIPRES web portal [40] using the program GARLI [41]. Each of our analyses employed a GTR + G + I model of nucleotide substitution, and parameters for this model were estimated during each run. For taxon-specific phylogenetics (see below), bootstrap analyses with 100 replicates were performed for each dataset using GARLI (with the above-described approach) or RAxML 7.2.8 Black Box [42]. Analyses using this latter program utilized default parameters with the exception that the proportions of invariant sites were estimated during the run. All phylogenies were visualized on the Interactive Tree Of Life (iTOL) website [43].

### Diagnostic PCR assays

Diagnostic PCR screening was used to assess the presence/absence of *Arsenophonus*, *Cardinium*, *Hamiltonella*, *Spiroplasma*, and *Wolbachia* across ants and lepidopterans. Screening for the former four symbionts ranged across our insect collections, while surveys for *Wolbachia* were more limited due to prior surveys of some of our targeted insects [26]. Nearly all screening assays were conducted on extractions from single individuals.

PCR results were only tallied for runs in which the positive controls amplified with the proper diagnostic primers and for which negative controls were clearly negative. Templates were scored positive when: 1) they yielded a product of the expected size in at least two separate reactions (for all samples), and (for all but two of our declared positives) 2) when BLASTn searches with the amplified sequences yielded a top hit to a bacterium from the expected taxon. Alternatively, templates were scored negative when they did not give rise to products of the expected size or if the top BLASTn hit was to a bacterium from outside the targeted clade (e.g. for three butterfly species with microbes BLAST'ing to *Providencia* instead of *Arsenophonus*). Those giving rise to non-repeatable amplification were scored as ambiguous and were not included in our frequency/incidence estimates.

### Systematic review of symbiont incidence

**Figure 1:**
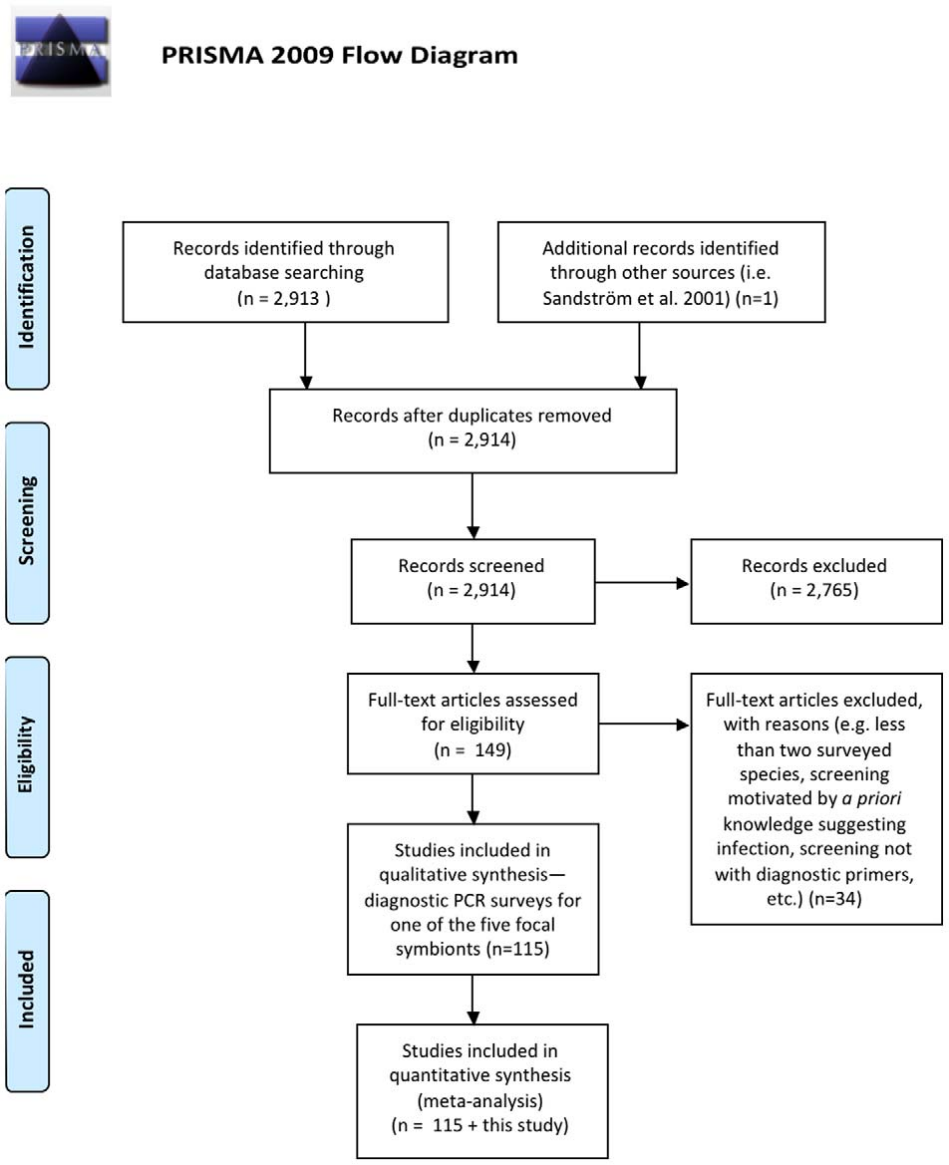
Study selection (PRISMA) flow diagram for our systematic review. Details on identification and selection of diagnostic PCR studies that surveyed for *Arsenophonus*, *Cardinium*, *Hamiltonella*, *Spiroplasma*, and *Wolbachia*.

To place our screening results into a broader context, we performed a systematic review of symbiont incidence across the arthropods (see Figure 1 for PRISMA flow diagram, and supplementary files for the PRISMA checklist). We began with a literature search, identifying published diagnostic PCR surveys for the five heritable symbionts targeted in this study. These studies were found through the Web of Science database by using the genus names of all five symbionts, separately, as queries. Literature searches were performed in 2009, and again in July 2011 and July 2012.

After scanning abstracts for terms indicative of molecular symbiont screening, the Methods sections of candidate papers were read to determine suitability. Only those using diagnostic PCR with species- or genus-specific primers were included. To avoid bias within individual studies we ensured that all species surveyed were fully listed (rather than only those with the symbiont) and that PCRs included negative controls.

For all suitable publications, we extracted data on the presence/absence of symbionts in individual arthropods (or pools of individuals) from tables, text, or supplementary files, compiling this into a spreadsheet that listed the names and taxonomy of surveyed arthropod species, and the presence/absence of the five symbionts screened for in this study. Our focus was limited to the species level, and we ignored data on intraspecific frequency when present. Since there was redundancy (i.e. screening of multiple individuals from some species within or across studies), we combined data so that species were declared positive if one or more individuals yielded a PCR positive in one or more of the included publications.

To reduce the effects of publication bias across studies, we excluded those that surveyed single host species since such papers are more likely to be published in the event of positive results. We also excluded studies with an obvious bias toward infection arising due to *a priori* knowledge of infection status or known reproductive manipulation before screening.

In total, the data used for our systematic review came from 115 publications and included 733 arthropod species surveyed for *Arsenophonus*, 1250 arthropod species surveyed for *Cardinium*, 585 arthropod species surveyed for *Hamiltonella*, 982 arthropod species surveyed for *Spiroplasma*, and 3994 arthropod species surveyed for *Wolbachia* (including our results). We graphed the proportions of infected species for all arthropod orders and families with at least 10 surveyed species, illustrating how symbiont frequencies vary across host taxa.

### Characterizing microbial communities through universal PCR and cloning

To pursue ambiguous diagnostic PCR results across 13 ant and two lepidopteran species, we cloned and sequenced 16S rRNA fragments amplified with either universal primers (9Fa and 1513R, from [44]) or primers targeting the Enterobacteriaceae (F40 and R1060) with a capacity to amplify 16S rRNA genes from *Arsenophonus* and *Hamiltonella* [45]. Additional sequencing of PCR products (direct or after cloning) amplified with universal 16S rRNA primers allowed us to study bacteria from seven ant species in the tribe Camponotini (hosts of obligate and heritable *Blochmannia* symbionts) along with two ant species harboring suspected *Wolbachia* infections. All newly generated 16S rRNA sequences were deposited in GenBank under the accession numbers KC136849–KC137148.

While few universal sequences were generated from moths and butterflies (12 sequences from one individual), we analyzed a total of 474 universal 16S rRNA sequences from 82 individual ants spanning 78 colonies and 74 species (203 sequences from 19 ants in this study, with the remainder coming from prior studies [44], [46], [47]). Phylogenetic analyses of these sequences allowed us to identify instances of heritable microbes across the ants (see Information S1 for more detail), providing a second means to survey for heritable symbionts.

Our analyses of 16S rRNA clone libraries also allowed us to assess the relative densities of heritable symbionts within their hosts (i.e. compared to co-inhabiting bacteria). To achieve this, all sequences generated with universal primers 9Fa and 1513R were classified using the RDP classifier [39]. The proportion of sequences classifying to heritable genera (i.e. *Blochmannia*, *Spiroplasma*, and *Wolbachia*) was then calculated for each infected host.

### Taxon-specific phylogenetic analyses

To further examine the phylogenetic placement of ant-associated bacteria related to heritable microbes, we performed four additional phylogenetic analyses on 16S rRNA sequences—one focusing on *Blochmannia* sequences and their relatives, one focusing on *Arsenophonus* and relatives, one focusing on other members of the Gammaproteobacteria, and one focusing on *Spiroplasma*. Related sequences identified through SeqMatch or BLAST searches were included in each of these analyses. Also included were representative sequences from microbes known to be heritable in various arthropods. Clustering with known heritable symbionts was used to suggest the potential for heritability among the novel bacteria identified from ants and lepidopterans.

### Wolbachia wsp sequence analyses

To study the diversity of *Wolbachia* strains infecting ants and lepidopterans, we sequenced the highly variable *wsp* gene for nearly all infected hosts. Chromatograms generated from direct sequencing were examined, and those with clean, single peaks were used to infer likely single infections. The *wsp* sequences from these single infections were submitted to GenBank (Accession #s below). Otherwise clean chromatograms that gave rise to multiple peaks at some positions were used to infer the presence of multiple *Wolbachia* strains in the targeted insects. *Wolbachia* from several of these hosts were subsequently studied by cloning and sequencing *wsp* fragments.

Sequences from *wsp* clone libraries were separately aligned using ClustalW on the EMBL-EBI website (http://www.ebi.ac.uk/Tools/msa/clustalw2/), and alignments were manually adjusted in MacClade [48]. Uncorrected pairwise distances were then computed for each alignment in PAUP v4.0b10 [49]. Distance matrices for each clone library were inspected to identify 99% phylotypes—groups of related sequences that were ≤1% divergent from all other members of their cluster and greater than 1% divergent from all sequences in other clusters. Using the same approach, 97% phylotypes were also identified for each library. To illustrate the diversity of *Wolbachia* strains found in single ant and lepidopteran hosts we calculated the number of phylotypes from each insect host and the number of sequence reads from each phylotype.

For each *wsp* library, one representative sequence per 99% phylotype was chosen for subsequent analyses and for submission to GenBank. Each of these representatives, and all of those from direct sequencing were queried against the *wsp* database on the *Wolbachia* MLST website (http://pubmlst.org/Wolbachia), identifying the closest relatives of the *wsp* alleles sampled in our study. Accession numbers for GenBank all *wsp* submissions were KC137149–KC137234.

## Results

### Infection across host species and populations—diagnostic PCR

Ants were frequently colonized by *Wolbachia* (28.6%), showing fewer associations with *Spiroplasma* (4.6%) and *Arsenophonus* (1.6%). Lepidopterans did not harbor *Arsenophonus*; but like the ants, these insects frequently possessed *Wolbachia* (24.7%), while few were colonized by *Spiroplasma* (2.5%). Neither *Cardinium hertigii* nor *Hamiltonella defensa* were found among any of the insects screened in this study (see Table 1 for summarized screening results and sample sizes, and Table S3 for details on all surveyed insects).

**Table 1:** Proportions of ants and lepidopterans testing positive for heritable symbionts in this study.

Symbiont	Ants††	Lepidopterans††
*Arsenophonus*	0.016 (n = 250)	0 (n = 256)
*Cardinium*	0 (n = 284)	0 (n = 164)
*Hamiltonella*	0 (n = 222)	0 (n = 251)
*Spiroplasma* †	0.046 (n = 364)	0.025 (n = 200)
*Wolbachia*	0.286 (n = 70)	0.247 (n = 158)

† *Spiroplasma* tallies were inferred in this study but were mostly derived from molecular screening in Funaro et al. 2011.

†† Numbers of surveyed species are indicated in parentheses.

*Wolbachia* prevalence varied extensively between army ant genera. Species from the genus *Aenictus* (subfamily Aenictinae) exhibited the highest infection rate, with 10 infected species out of the 12 that were surveyed. Only *Neivamyrmex* species were found to be infected among the five surveyed genera from the subfamily Ecitoninae, with all five surveyed species from this genus harboring *Wolbachia*. In contrast, *Wolbachia* were not found among 19 ant species from the genus *Dorylus* (subfamily Dorylinae). Among well-sampled lepidopteran families (i.e. n≥10 species surveyed), Hesperiidae species were the most commonly infected by *Wolbachia* (7/15), while the Riodinidae had the lowest infection frequency (0/11).

Eleven of the 46 surveyed army ant species were sampled with intraspecific replication, and among these only one of the five infected species showed evidence for intraspecific polymorphism (see Table S4 for intraspecific screening results). Intraspecific *Wolbachia* surveys across colonies from the ant genus *Polyrhachis* (tribe: Camponotini) also showed 100% infection based on small samples from the two infected species (N = 5 for *P. flavibasis* and N = 3 for *P. rufifemur*).

Looking more broadly across the *Polyrhachis* genus, *Wolbachia* symbionts were found in 5/24 (20.83%) newly screened species. Interestingly, six out of 29 *Polyrhachis* species were found to harbor *Spiroplasma* (20.7%), an incidence that was significantly greater than that found for the remaining ants (10/338, or 3.0%) (2-tailed Fisher's Exact Test, p = 0.0007). In contrast, our surveys detected *Spiroplasma* in 0/11 camponotines outside of *Polyrhachis*, hinting at unique enrichment within this particular ant genus. When we combined our *Wolbachia* screening data with those from prior *Polyrhachis* surveys [26], [50], [51], it became clear that the incidence of *Spiroplasma* was actually comparable to that of *Wolbachia* within this host genus (20.7% of 29 species vs. 20.6% of 34 species).

Among the *Polyrhachis* species possessing *Spiroplasma*, only one was subjected to screening beyond a single individual or colony. In this case, the bacterium was found in just one worker across seven surveyed colonies (n = 1 surveyed worker per colony), deviating from the trend of high intraspecific prevalence for ant-associated *Wolbachia*. Intraspecific screening for *Arsenophonus* suggested that low frequency infections may be common for these microbes, as well. For instance, *Arsenophonus* symbionts were detected in one out of two surveyed males from the ant *Nomamyrmex esenbeckii*, but in none of the workers sampled from four separate colonies of this species. Similarly, in an unidentified *Labidus* species, one out of two surveyed males was infected. In the third army ant found to host *Arsenophonus* (*Eciton burchellii*), this symbiont was found in just one worker among those from seven sampled colonies.

### Infection across the arthropods —a systematic review of diagnostic screening studies

**Figure 2:**
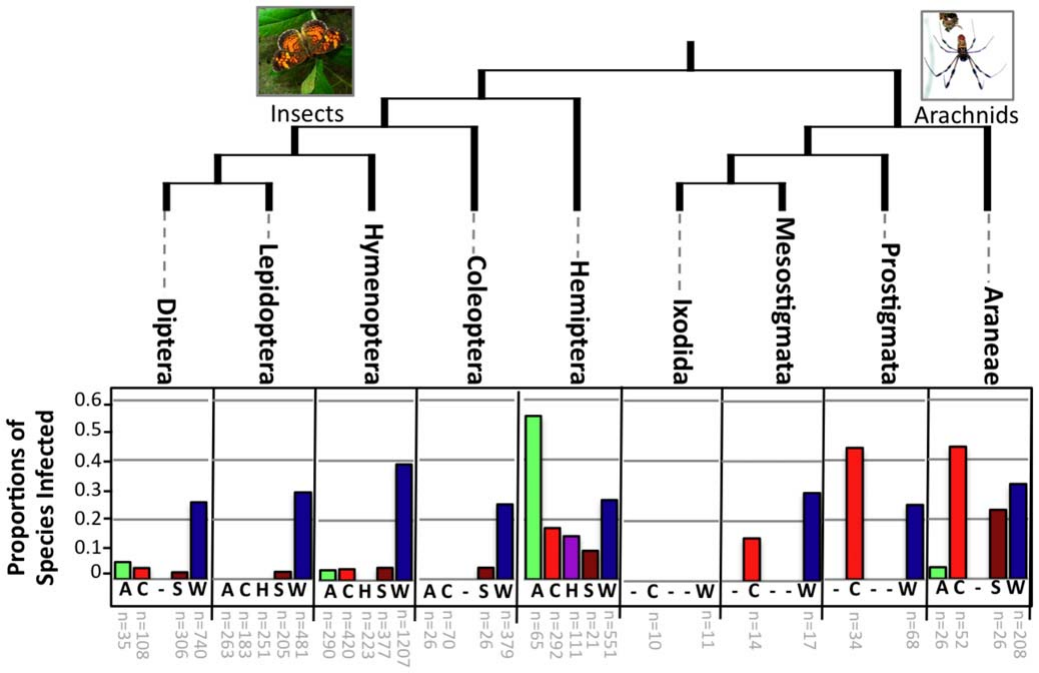
Frequencies of *Wolbachia* across arthropod orders. Proportions of infected species for all orders with n>10 species surveyed for *Arsenophonus* (A), *Cardinium* (C), *Hamiltonella* (H), *Spiroplasma* (S), and/or *Wolbachia* (W). “−” symbols indicate cases where data were not illustrated due to sample sizes of less than 10 surveyed species. Numbers of surveyed species are indicated below each respective bar. Data used for this figure were generated with diagnostic PCR surveys and are presented in Table S5. The phylogeny was drawn from information on the Tree of Life Website (tolweb.org). Only those orders with at least two symbionts meeting the above criteria are illustrated.

To place our findings from ants and lepidopterans into context, we summarized the frequencies of the five focal symbionts—*Arsenophonus*, *Cardinium*, *Hamiltonella*, *Spiroplasma* and *Wolbachia*— across 4,366 arthropod species based on the results from 115 diagnostic PCR screening studies (Table S5). Infection frequencies within the insect orders Coleoptera, Diptera, Hymenoptera, and Lepidoptera were found to mirror the results from our diagnostic screening, with ∼20–35% of species infected by *Wolbachia* and ∼6% or less harboring *Arsenophonus*, *Cardinium*, and *Spiroplasma* (Figure 2). The Lepidoptera exhibited no instances of infection with any symbionts except *Spiroplasma* (2.4% of species infected) and *Wolbachia* (29.1% species infected), again matching results from our screening.

The Hemiptera deviated from other insect groups, showing higher instances of *Arsenophonus*, *Cardinium*, and *Spiroplasma*. Outside of the insects, arachnids were common hosts of *Cardinium*, with frequencies ranging from 14–44% outside of the Ixodida (ticks), which showed 0% infection in the targeted studies. The Araneae (spiders) were especially enriched for *Spiroplasma* (23.1% of species infected) in addition to *Cardinium* and *Wolbachia* (44.2% and 31.7% infected species, respectively).

**Figure 3:**
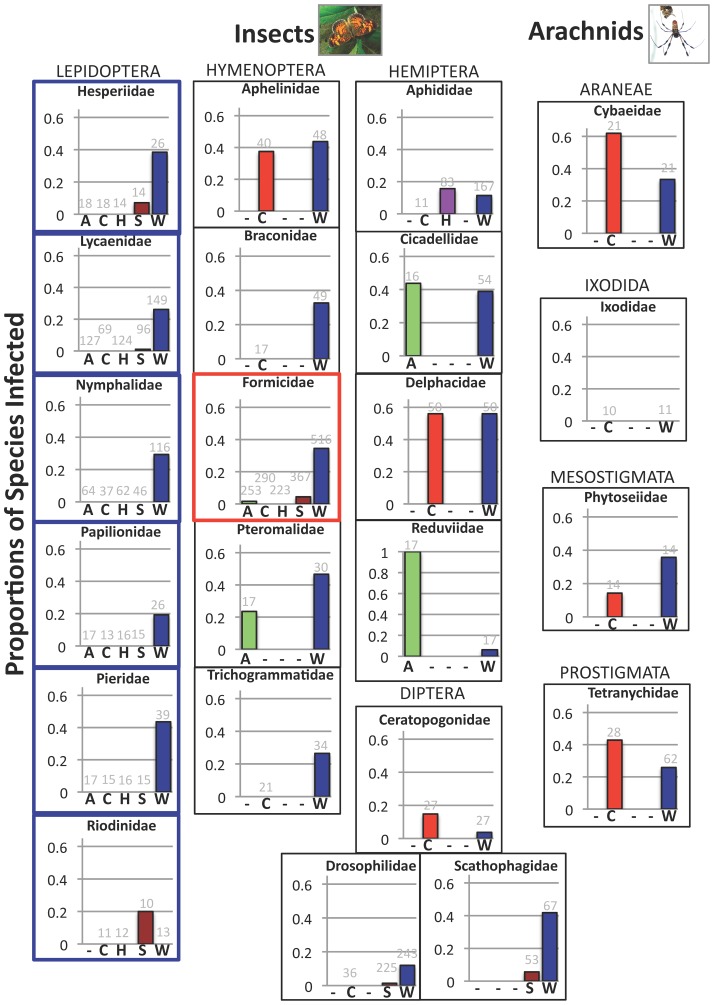
Frequencies of *Wolbachia* across arthropod families. Proportions of infected species for all families with n>10 species surveyed for Arsenophonus (A), Cardinium (C), Hamiltonella (H), Spiroplasma (S), and/or Wolbachia (W). “−” symbols indicate cases where results were not illustrated due to sample sizes of less than 10 surveyed species. Families of butterflies and moths were heavily sampled in this study and are highlighted in blue. Infection frequencies within the ant family Formicidae are highlighted in red. Names of arthropod orders are listed in capital letters above the data for their respective families. Numbers of surveyed species are indicated above each respective bar. Data used for this figure were generated with diagnostic PCR and are presented in Table S5. The phylogeny was drawn from information on the Tree of Life Website (tolweb.org). Only those families with at least two symbionts meeting the above criteria are illustrated.

Examination of symbiont prevalence within arthropod families (with n ≥ 10 sampled species) provided further insight into the taxa shaping trends seen at the ordinal level (Figure 3). For instance, within the Hemiptera *Arsenophonus* were enriched within the Cicadellidae (leafhoppers) and Reduviidae (specifically, the kissing bugs), though they were not extensively surveyed across other hemipteran families. And although *Cardinium* appeared common among the Hemiptera, this largely resulted from high prevalence within the Delphacidae (planthoppers), compared to lower infection rates within the Aphididae (based on limited screening). Across well-sampled Hymenoptera families, *Cardinium* were only found in the Aphelinidae, while findings of *Cardinium* among dipterans were confined to species from the Ceratopogonidae (biting midges).

Variation in *Wolbachia* prevalence also existed among genera from the same families, most notably within the Aphididae (aphids), Culicidae (mosquitoes), Tephritidae (true fruit flies), and Formicidae (ants) (Table S6). In the former case, *Wolbachia* were found in only 10% of species from the genus *Aphis* (n = 30) compared to 50% of the surveyed *Cinara* species (n = 14). Within the Tephritidae, infection ranged from 29.5% (n = 44) of species from the genus *Bactrocerra* to 100% (n = 10) within the genus *Anastrepha*. Among the mosquitoes, the genus *Anopheles* had no infected species out of 35 surveyed, while the genera *Aedes* and *Culex* had *Wolbachia* infection rates of 26.5% (n = 34) and 50% (n = 34), respectively. Finally, the ant family Formicidae had infection frequencies ranging from 0% (*Dolichoderus* and *Dorylus* with n = 15 and n = 22 surveyed species, respectively) to over 80% within the genera *Tetraponera* (n = 10), *Aenictus* (n = 16), and *Formica* (n = 10).

It should be mentioned that our criteria for the inclusion of symbiont screening data (i.e. diagnostic PCR surveys across two or more species) led to exclusion of several confirmed infections. In fact, some of the hosts with 0% reported infection in our figures and tables are known to harbor the microbes in question. This is true for ticks (Ixodida), for example, which harbor *Arsenophonus* [52]. It is also true for ants (Formicidae), where at least one species is known to harbor *Cardinium* [53].

### Infection across ant and lepidopteran species—universal PCR and phylogenetic analysis

While diagnostic screening permitted surveys across a diverse range of ants, moths, and butterflies, sequencing of universal 16S rRNA PCR products provided a second molecular approach for symbiont surveys across the ants (see Table S7 for summary statistics of clone libraries and a list of sampled species). Based on this dataset, RDP classification revealed that *Wolbachia* were present in 28/74 sampled ant species. This same approach identified heritable *Blochmannia* symbionts in all but one of nine sampled species from the tribe Camponotini. *Spiroplasma* symbionts were found in two of these nine camponotine species (genus *Polyrhachis*), and in two of the 65 other ant species surveyed with the universal approach. Aside from potentially heritable symbionts from the Enterobacteriaceae (see below), universal PCR and sequencing did not identify maternally transmitted bacteria from other common groups (e.g. *Rickettsia* sp., *Hamiltonella defensa*, *Serratia symbiotica*, *Regiella insecticola*, *Cardinium hertigii*, etc.) either from ants or from the one butterfly sampled with universal primers.

**Figure 4:**
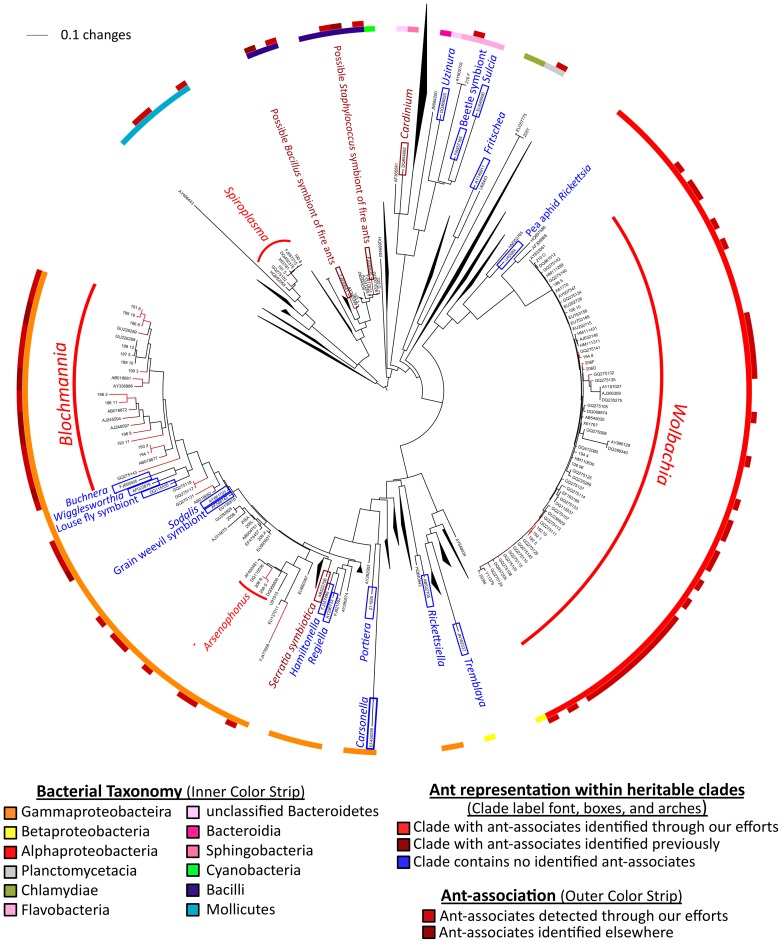
16S rRNA phylogeny of known heritable symbionts and microbes from ants. Maximum phylogeny showing relatedness between ant associates and known maternally transmitted bacteria, thus illustrating the range of candidate heritable symbionts across the ants. Lineages of heritable symbionts are labeled and color-coded to indicate the presence of ant-associates. Color strip circles are used to indicate bacterial taxonomy (inner circle) and ant-association (outer circle). For ease of viewing, several clades on the original phylogeny were collapsed (i.e. those without heritable symbionts). The full tree is presented in Figure S1. Note that lifestyle heterogeneity within *Spiroplasma* and *Arsenophonus* lineages means that identified ant-associates are not certain to be heritable. Also note that the two “possible” symbionts of ants (a *Staphylococcus* sp. and a *Bacillus* sp.) were both detected in hemolymph and in eggs laid by queens, suggesting heritability.

Overall, ants harbored heritable symbionts from six, and possibly eight, lineages of heritable symbionts (*Arsenophonus*, *Blochmannia*, *Spiroplasma*, and *Wolbachia*—shown here; *Cardinium hertigii* and *Serratia symbiotica*—shown elsewhere [53], [54]; and two candidate symbionts from fire ants [55]; see Figures 4 and S1). The presence of bacteria clustering within clades of other heritable bacteria from the Enterobacteriaceae (Figure S2) suggests that this number could be even higher. These results contrast with what we see in the Lepidoptera, with only two heritable lineages (*Spiroplasma* and *Wolbachia*) being reported to date.

### Taxon-specific phylogenetic analyses

**Figure 5:**
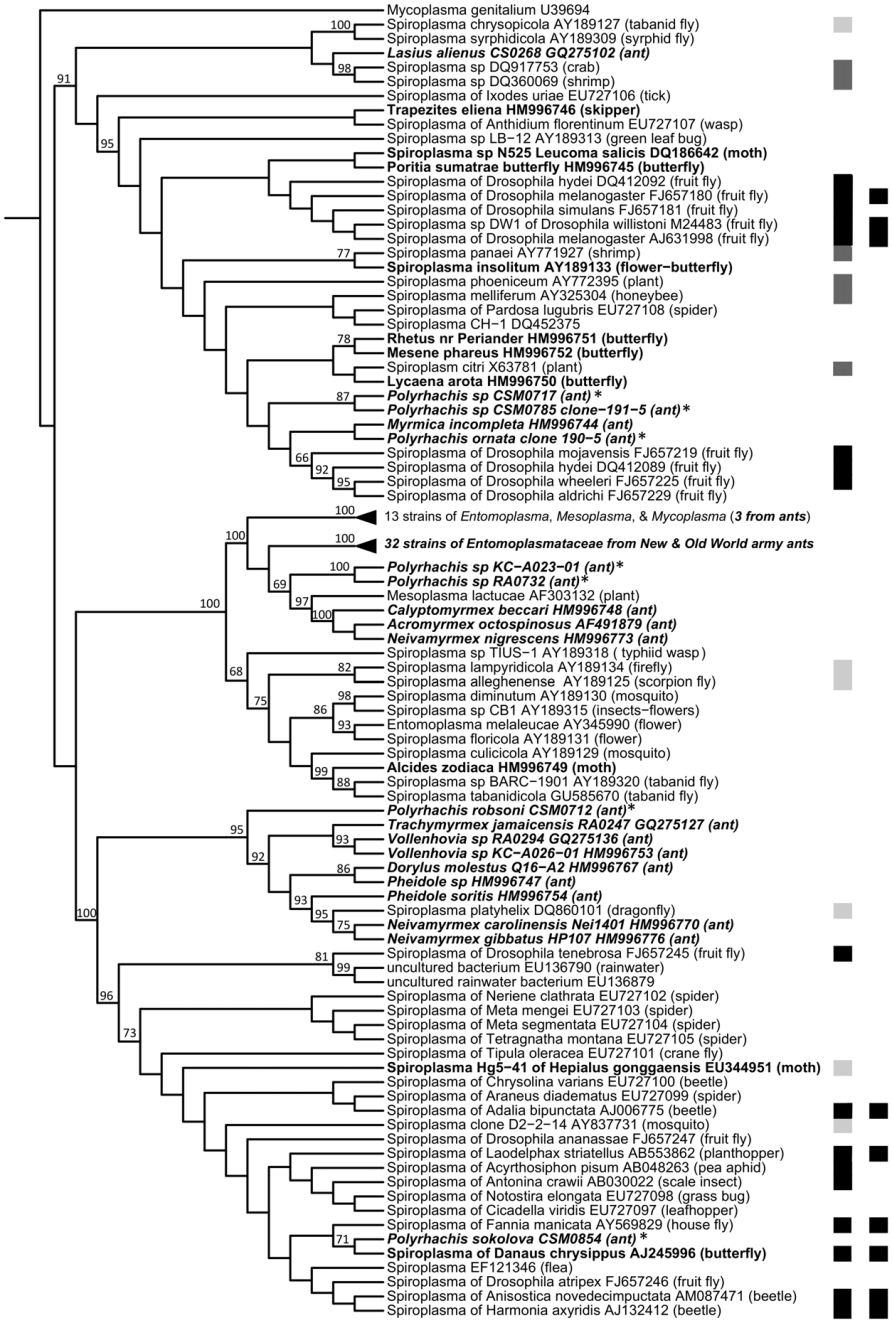
16S rRNA phylogeny of *Spiroplasma* from ants, lepidopterans, and other hosts. Maximum likelihood phylogeny illustrating relatedness between ant associates and various *Spiroplasma* symbionts. The inner-most color strip illustrates known lifestyle, with gut associates being presented in light gray, pathogens in dark gray, and heritable bacteria in black. In the outer strip, the phenotype of male-killing is illustrated. Microbes are named after their hosts. Those from ants are in bold and italics; those from lepidopterans are in bold font; and those reported from *Polyrhachis* in this study are indicated with an asterisk. Non-*Spiroplasma* clades are collapsed for brevity. Bootstrap values greater than 60 are indicated above their respective nodes.

Maximum likelihood phylogenies showed that *Arsenophonus* (Figure S4) and *Spiroplasma* (Figure 5) from ants clustered with heritable bacteria from other ant or arthropod hosts, although several ant-associated *Spiroplasma* grouped into lineages without known heritable relatives. As seen in our universal 16S rRNA phylogeny (Figure 4), a number of other ant-associates (identified with universal 16S rRNA PCR primers) clustered within a gammaproteobacterial clade comprised almost entirely of insect-associated symbionts (Figure S2), including heritable bacteria from tsetse flies, scale insects, psyllids, and aphids, as well as gut bacteria from ants. Among these, several were related to coevolved *Blochmannia* symbionts from carpenter ants (Figure S3). Although the long branches separating ant-associates from known symbionts prevent us from making stronger conclusions, these Enterobacteriaceae microbes could potentially be heritable symbionts or specialized residents of ant guts.

### Within-host infection trends—relative symbiont densities

When considering hosts infected with microbes from vertically transmitted clades, we found a tendency for heritable symbionts to dominate bacterial communities (Table S7). In *Camponotus* and *Polyrhachis* species, *Blochmannia* (an essential heritable symbiont) comprised a median of 50% of the universal sequence reads across eight worker ants from seven species (with n≥4 sequence reads each). In two *Polyrhachis* species, sequence reads BLAST'ing to *Spiroplasma* comprised 50% and 57.1% of our clone libraries, respectively equaling and exceeding the numbers of *Blochmannia* reads in the same hosts. Interestingly, in the one camponotine with *Wolbachia* and n≥4 sampled sequences, 9/9 reads belonged to this bacterium.

Looking across all ants with n≥4 sequence reads, *Wolbachia* made up anywhere from 20% to 100% of sequence libraries when present (n = 6 sampled ant species), with a median representation of 68.33%. In addition, clean *Wolbachia* sequences have been generated from direct sequencing of 20 other universal 16S rRNA products from different ants (two new to this study), suggesting high abundance compared to co-infecting microbes within the same hosts. When we consider that *Wolbachia* were detected in 37.8% (28/74) of the ant species surveyed with universal primers, and that 16.5% (78/474) of all universal sequence reads came from *Wolbachia*, it is clear that this symbiont is a dominant member of microbial communities within many ants.

### Within-host infection trends—Wolbachia co-infection

Prior research suggested that *Wolbachia* communities from individual insects may themselves consist of multiple strains [28], hinting at even more diversity within hosts than can be appreciated through 16S rRNA analyses. In this study we generated 32 clean *wsp* sequence reads (i.e. no multiple peaks) from *Wolbachia*-infected Lepidoptera out of direct sequencing attempts from 35 species. Multiple peaks within the remaining chromatograms suggest that roughly 8.6% of lepidopteran infections involved more than one strain. Among the *Polyrhachis* ants that were newly discovered to have *Wolbachia*, we had difficulty generating quality *wsp* sequences. Instead, we sampled two MLST genes, *gatB* and *hcpA*, observing that 6/8 samples (from five ant species) likely harbored multiple *Wolbachia* strains. Sequencing of *wsp* from *Wolbachia* in *Aenictus* and *Neivamyrmex* army ants identified eight likely multiple infections out of direct sequencing attempts from 22 different workers (spanning 13 species, with direct sequencing from 1–5 workers per species).

To better assess the diversity of *Wolbachia* within ant and butterfly hosts, we targeted several of those yielding multiple chromatogram peaks in prior direct sequencing attempts [26]. The *wsp* gene was amplified, cloned, and sequenced from 15 ants and two lycaenid butterflies, with an average of 8.6 reads per individual ant (range: 3–22) and 4.5 per butterfly (range: 4–5). Among the two *Wolbachia wsp* libraries from butterflies, two phylotypes were detected from the host with a suspected multiple infection, while just one phylotype was found in a host with a suspected single infection (true for both 99% and 97% phylotype criteria). In only two ant libraries did we detect just one *wsp* variant (99% and 97% phylotype criteria): the first of these had been selected *a priori* due to its suspected single infection status, while the other was sampled at low depth (n = 3 reads). As such, 13 out of 14 suspected instances of multiple *Wolbachia* infection were confirmed (99% phylotype results; see Table 2).

**Table 2:** The diversity of *Wolbachia* strains from single insect hosts as inferred through sequencing of *wsp* libraries.

Common host name	Host genus and species	Total # of phylotypes†	# reads in phylotype 1	# reads in phylotype 2	# reads in phylotype 3	# reads in phylotype 4	# reads in phylotype 5	# reads in phylotype 6	Total # sequence reads
Ant	*Aenictus* sp. 2	2	5	1					6
Ant	*Cardiocondyla emeryi*	2	4	2					6
Ant	*Formica wheeleri*	3	3	3	7				13
Ant	*Formicoxenus provancheri*	4	4	2	1	4			11
Ant	*Formicoxenus provancheri*	2	2	1					3
Ant	*Heteroponera microps*	2	2	2					4
Ant	*Labidus spininodis*	4	1	2	1	3			7
Ant	*Megalomyrmex latreillei*	6	5	2	12	1	1	1	22
Ant	*Myopias lobosa*	3	4	1	1				6
Ant	*Myrmelachista* sp.	1	3						3
Ant	*Neivamyrmex nigrescens*	3	13	1	3				17
Ant	*Odontomachus* sp.	3	1	6	1				8
Ant	*Pheidole umphreyi*	3	2	1	1				4
Ant	*Rhytidoponera metallica*	1	9						9
Ant	*Temnothorax tricarinatus*	5	4	2	1	1	2		10
Butterfly	*Jamides alecto*	1	4						4
Butterfly	*Madeleinea sigal*	2	4	1					5

† Phylotypes described based on 99% sequence similarity, as described in the methods.

When using 99% *wsp* sequence identity as the cut-off for phylotype assignment, the median number of strains detected per studied ant with suspected multiple infection was three. One worker of the species *Megalomyrmex foreli* harbored six different *Wolbachia* strains. Five strains were detected in a single *Temnothorax tricarinatus* worker, while single workers from two other species (*Labidus spininodis* and *Formicoxenus provancheri*) each harbored four different strains. Results were nearly identical when using the 97% criterion—there were 40 detected strains using this measure across all 14 ants with suspected multiple infections, compared to 43 with the 99% criterion.

We should note that the existence of singleton OTUs in several of the sampled libraries leaves open the possibility that either sequencing error or PCR artifacts (i.e. chimeras) were responsible for a small proportion of the detected diversity. Indeed, two alleged strains out of the 40 detected from ants (based on the 97% criterion) appeared to harbor *wsp* alleles that were likely recombinants of others from the same host. While these could represent examples of novel allele generation facilitated by multiple infections, they could also have stemmed from chimeric PCR products. Aside from these instances, it is unlikely, however, that non-recombinant singleton sequences with over 3% divergence from others in the same host arose frequently due to sequencing or PCR error.

### Trends of host-association inferred from wsp alleles

To ascertain whether *wsp* alleles from co-infecting strains showed similar trends of host association as seen for singly infecting *Wolbachia* strains of ants [26], [56], a representative sequence from each 99% phylotype was used to perform a query search against the *Wolbachia* MLST database (http://pubmlst.org/wolbachia/). Eleven *wsp* sequences were identical to alleles in the database (Table S8). All of these were derived from *Wolbachia* strains of ants, with only one (allele 18) that was also found outside of ants (in lepidopterans). A slim majority (23/42) of the remaining *wsp* alleles from multiply infected ant hosts had highest similarity to those from ant-associated *Wolbachia* strains established as single infections. Alleles from New World ants were most similar to those from *Wolbachia* found in various New World locations. And those from ants in the Old World and Oceania regions were, likewise, most similar to *wsp* alleles of *Wolbachia* from ants in these same regions. Trends from army ants with single *Wolbachia* infections largely mirrored those described above. Similarly, 17/35 *wsp* alleles from lepidopterans were most closely related to those found previously in other moths and butterflies (Table S8).

## Discussion

### Microbial menageries—players within bacterial communities from ants and lepidopterans

Over the past decade, the cataloging of microbial communities across the insects has begun to accelerate. Long-known to play important nutritional roles in groups such as termites [57], [58] and sap-feeding hemipterans [59], more recent discoveries also hint at common microbial roles in defense against natural enemies [33]–[36], [60] and tolerance of abiotic conditions [61]–[63]. Although metagenomic approaches can provide powerful insight into the functions of microbial communities [64], [65], surveys of animal-associated bacteria often lack the ability to determine lifestyles and functions of the identified bacteria and fungi. It is partially for this reason that surveys for widespread microbes with conserved lifestyles—like those employed here—are especially useful, as they enable researchers to identify symbionts with a range of plausible phenotypic effects.

In this study we have characterized microbial associates of two diverse and important groups of insects, looking broadly across the ants while focusing mostly on the Lycaenidae and Nymphalidae families within the Lepidoptera. Ants, moths, and butterflies are found across most terrestrial habitats and can variably function as herbivores, generalists, and predators; as pollinators and seed dispersers; as contributors to nutrient recycling; as defensive mutualists of plants and plant pests; and as major plant pests themselves through their abilities to defoliate vegetation. Symbiotic microbes could play roles in several of these functions, while shaping other lifestyle attributes that influence the fate of their host species.

Microbial communities of ants and lepidopterans have been assessed previously, though typically with less depth and breadth. For instance, gut bacteria of lepidopteran larvae and adults have been documented for a small number of species [66]–[68], while recent studies have also chronicled the distributions and evolution of bacteria from the guts [46], [69]–[71] and cuticles [72], [73] of various ants. Such research is of interest given the hypothesis that gut bacteria have facilitated the evolution of herbivory across the ants [44], and also given their proposed use [74], or actual function [66], in biological control. Studies on these insects have also focused on heritable symbionts. Facultative heritable symbionts such as *Wolbachia*, *Spiroplasma*, *Cardinium*, *Serratia*, and *Arsenophonus* have been documented from ants [47], [53], [54], [75]–[78], with only the former two groups being reported from lepidopdterans [79], [80].

When combined with our findings, it appears that the Lepidoptera harbor a more limited array of common heritable symbionts (thus far, just two), when compared to the ants, for reasons that are currently unknown. While we estimate six species of heritable symbionts across the ants, this number may be higher. For instance, fire ant queens harbor bacteria in their hemolymph that are also found in eggs and ovaries [55], while other ants harbor bacteria from a symbiotic clade within the Gammaproteobacteria (Figure S2). More work is needed to elucidate the lifestyles of these bacteria, to explore the possibility of non-canonical or rare heritable symbionts across ants and lepidopterans, and to confirm the lifestyles of *Arsenophonus* and *Spiroplasma*, which come from lineages where heritability is not ubiquitous [81].

It should be noted that while our screening assays could have plausibly missed some of the targeted bacteria (e.g. due to primer-annealing site mismatches), screening and sequencing with universal 16S rRNA primers across several dozen ants identified no candidate heritable bacteria other than *Blochmannia* and those detected through diagnostic screening. For this reason, our findings that *Cardinium* and *Hamiltonella* are rare across the ants are unlikely to be the result of methodological bias. This rarity is at least mildly surprising, in light of the frequent trophic interactions between ants and insects known to host such bacteria (i.e. aphids and whiteflies).

Similar to our growing, but limited, knowledge of microbial diversity in the insects targeted here, our understanding of the functions and consequences of identified bacteria is also in the budding stages. A few exceptions can be seen for lepidopterans, in which *Wolbachia* can manipulate reproduction [79], [80], [82]. Among the ants, members of the tribe Attini are known to harbor antibiotic-producing bacteria on their cuticles that help to defend their fungal gardens [83], while those from the Camponotini harbor nutritional *Blochmannia* symbionts in specialized cells of their midgut [84]–[86]. Aside from indirect evidence for nutritional roles of gut symbionts in herbivorous ants [44], [70], the vast majority of the remaining bacteria described thus far from ants remain uncharacterized, including many related to free-living microbes detected from ants in our own work (Figure S1).

Certainly, then, discoveries of heritable symbionts with a potential for nutrition, defense, or reproductive manipulation [1] suggest areas for investigation. Our findings suggest that such roles should be especially studied for *Wolbachia*, given their notable prevalence and infection patterns among the insects studied here. Currently, *Wolbachia* are known to induce male-killing (MK), feminization, and cytoplasmic incompatibility (CI) in lepidopterans [80], [82], [87], [88]. But while CI and MK are consistent with patterns from *Wolbachia*-infected ants [89], we know little about the explicit effects of these bacteria on their ant hosts [28], [90], [91]. Like *Hamiltonella* [35], *Spiroplasma* [34], [60] and possibly *Arsenophonus* [32], *Wolbachia* can also defend some insects against natural enemies [33], [36], raising an unexplored possibility for the ants and lepidopterans.

### Wolbachia steal the show…but not on all stages

In addition to characterizing the heritable microbial communities of ants, butterflies, and moths, our work synthesizes the current state of knowledge on the distributions and frequencies of these bacteria across the arthropods. While a good deal was previously known about these distributions, prior efforts had not approached the depth or breadth of sampling attained here within insect taxa. Our screening efforts and systematic review, therefore, facilitate some of the broadest and most comprehensive examinations of 1) variation in symbiont prevalence across host taxa and 2) the diversity of symbionts within well-sampled host taxa. In turn, these examinations allow initial glimpses of the correlates of infection—both phylogenetic and ecological—which may suggest causes and consequences of symbiont proliferation.

As noted above, our results indicate that *Wolbachia* are by far the most prevalent heritable symbionts found across the ants and within some butterflies, followed by *Spiroplasma* (both) and *Arsenophonus* (only in ants). Only one surveyed host group among these organisms, provided an exception to this rule, with the genus *Polyrhachis* showing a high prevalence of *Spiroplasma*. However, the origination of most targeted *Polyrhachis* species from one general location (i.e. the Australian Wet Tropics) suggests a need for further studies across a broader geographic range. Furthermore, four of the six identified *Polyrhachis* infections came from species in the subgenus Chariomyrma (4/6 species infected, compared to 2/17 in species from other subgenera). This indicates that *Spiroplasma* enrichment could be a specific feature of these close relatives, rather than a genus-wide attribute.

Similar to our results for the focal insects, *Wolbachia* appears to be the predominant heritable symbiont among the Coleoptera, Diptera, and Hymenoptera. Yet *Wolbachia* dominance is not seen across all arthropod orders. Notably, some groups within the Hemiptera show a pronounced enrichment for *Arsenophonus*, while several groups of arachnids are enriched for this bacterium and/or *Cardinium*, trends that have generally been recognized through prior reports [76], [92]–[94].

It is important to note that most of the sub-taxa within the different orders and families highlighted in Figures 2 and 3 have not been sampled with sufficient depth. In fact, trends gleaned for high *Arsenophonus* and *Cardinium* frequencies among the arachnids are based largely on in-depth sampling within single families in the Prostigmata (mites), Mesostigmata (mites), and Araneae (spiders). And while the Hemiptera have been sampled with greater depth and breadth for some symbionts, high incidence of *Arsenophonus* is driven by results from only two families (Cicadellidae and Reduviidae), while high *Cardinium* levels are promoted by frequent infection in the Delphacidae. So given that related taxa can differ drastically in symbiont infection frequencies (e.g. Table S6), it is clear that apparent differences in symbiont prevalence across host taxa should be interpreted cautiously until a larger portion of diversity from lower-level taxa can be sampled.

But even after accounting for sporadic and/or limited sampling of different groups, *Wolbachia* is nevertheless dominant amongst the heritable symbionts—a result that has been appreciated previously on smaller scales [17], [19], [21]. This raises the question of why *Wolbachia* symbionts have been so much more successful in obtaining broad host ranges and high incidence. Possibilities include their capacity for rapid molecular evolution through recombination or perhaps their abilities to persist in many types of hosts due to their wide range of conferred phenotypes (discussed further in Information S1).

### Side-shows or show-stealing stars?—What host factors govern symbiont prevalence?

While *Wolbachia* are the most prevalent among the heritable symbionts found across the arthropods, this obscures the fact that their frequencies vary between various host groups, something seen also for less prevalent and more host-range-restricted bacteria such as *Hamiltonella*, *Cardinium*, and *Arsenophonus*. This indicates that phylogeny is a correlate of prevalence for several symbionts (see also Table 3). Such a trend could extend from multiple symbiont acquisitions by a particular host group, or a limited number of acquisitions followed by codiversification, a scenario proposed for *Arsenophonus* and their blood-feeding hosts [76].

**Table 3:** Prevalence, ranges of infected hosts, and correlates of symbiont prevalence as inferred from our systematic review.

	*Distributional Trends*		
Symbiont	Prevalence and Host Range	Host Taxonomy	Life History†
*Arsenophonus*	% species infected: 7.50 (n = 733)	Enriched among some hemipterans, including leafhoppers and kissing bugs	Enriched among unrelated blood-feeding arthropods
	# infected arthropod families: 18 (n = 113)		
	# infected arthropod orders: 6 (n = 16)		
	Dominant symbiont within 1 of 10 well-sampled arthropod orders		
*Cardinium*	% species infected: 9.28 (n = 1250)	Enriched among mites and spiders	Enriched among some unrelated haplodiploids (mites, armored scale insects, parasitic wasps)
	# infected arthropod families: 22 (n = 191)		
	# infected arthropod orders: 9 (n = 29)		
	Dominant symbiont within 3 of 10 well-sampled arthropod orders		
*Hamiltonella*	% species infected: 2.74 (n = 585)	Enriched among aphids	No identified trends
	# infected arthropod families: 3 (n = 19)		
	# infected arthropod orders: 1 (n = 3)		
	Dominant symbiont within 0 of 10 well-sampled arthropod orders		
*Spiroplasma*	% species infected: 3.77 (n = 982)	Enriched among *Polyrhachis* ants from the subgenus *Chariomyrma* from the Australian Wet Tropics; rare among related *Camponotus* ants	No identified trends
	# infected arthropod families: 15 (n = 95)		
	# infected arthropod orders: 6 (n = 14)		
	Dominant symbiont within 0 of 10 well-sampled arthropod orders		
*Wolbachia*	% species infected: 31.25 (n = 3994)	Enriched among *Aenictus* army ants; rare among *Dorylus* army ants	Rare in two groups of cyclical parthenogens (aphids and oak gall wasps)
	# infected arthropod families: 183 (n = 356)	Enriched among *Culex* mosquitoes; rare among *Anopheles* mosquitoes	Possibly more common among ant species with dependent colony founding, with exceptions in some groups.
	# infected arthropod orders: 24 (n = 38)		
	Dominant symbiont within 5 of 10 well-sampled arthropod orders		

† Focusing on traits found in two or more unrelated groups enriched for the given symbiont.

In addition to phylogenetic correlates of symbiont prevalence, similar biological attributes can be found in some unrelated hosts enriched for the same symbionts, including diet (i.e. blood-feeing, for *Arsenophonus*), genetic system (i.e. haplodiploidy, for *Cardinium*), and modes of ant colony founding (i.e. dependent-founding, for *Wolbachia* [28], [51]). While exceptions to these biological correlates reveal a need for further examination, these trends do suggest hypotheses for symbiont function and for host attributes favoring the spread of facultative symbionts (see Table 3 for an overview, and Information S1 for more details).

### A profile of the ants' symbiotic menageries

In addition to our focus on bacterial distributions across host taxa, we have also characterized features of heritable symbionts that are evident at other hierarchical levels. To summarize: 1) ant hosts commonly harbor multiple *Wolbachia* strains within single workers; 2) heritable symbionts are abundant, if not dominant, members of ants' microbial communities (resembling trends from some [95]–[97], but not all [98] prior studies); 3) *Wolbachia* may be found at high frequencies within some ant species (raising questions about the prevalence of loss from adult workers [89], [90], [99]); and 4) heritable symbionts from related hosts appear closely related themselves, suggesting host specificity. While these observations are not entirely novel, the scope of our surveys does newly allow for generalizations across the ants. Below, we finish with a brief discussion of one of these findings.

### High levels of multiple infection across the ants—trends and implications

While analyses of 16S rRNA genes allow us to decipher the varieties of bacterial species from animal hosts, sequencing of more rapidly evolving genes is needed to distinguish among related strains co-infecting the same hosts. Interestingly, such efforts in this study make it clear that ants are especially predisposed toward co-infection with multiple strains of *Wolbachia*. This trend had been seen before in a limited range of wood ants, fire ants, and leaf-cutter ants [89], [100], [101]. Furthermore, in a previous study spanning a broader range of the Formicidae, we observed that 37.9% (33/87) of *wsp* chromatograms from ants had multiple peak patterns suggestive of multiple *Wolbachia* infections. In contrast, this total was considerably lower for surveyed butterflies (16.7% from 3/18 species with *wsp* sequence confirmation) [26]. When combined with chromatogram observations here, the number of *Wolbachia* infections estimated to consist of two or more strains reaches 40.2% (47/117) for the ants and 11.3% (6/53) for lepidopterans. The methodological basis for these estimates becomes more sound when we note that all but one of 15 examined multiple infections inferred from chromatogram viewing was confirmed through *wsp* cloning and sequencing in this study (Table S4), with a median number of 3 strains per multiply infected host. While insects such as lice and tephritid fruit flies have been found to harbor multiple *Wolbachia* strains [102], [103], up to eight strains have been detected within single worker ants, revealing a high diversity of these symbionts at the individual level [100].

So what are the implications of multiple infections? Clearly these should provide an avenue for the exchange of genetic material. Could ants, then, serve as a common group of melting pot hosts, facilitating genetic exchange among various *Wolbachia* lineages [28]? Such a role would be most far-reaching if multiply infected ants served as a cross-roads for generalist *Wolbachia* strains coming from numerous host taxa. Yet our inferences from *wsp* relatedness do not suggest this to typically be the case. Instead, we find that the *wsp* alleles sampled in multiply infected ants are most often related to those of strains grouping into ant-specific lineages on MLST phylogenies (Table S8). While we recognize the limitations of *wsp* as a phylogenetic marker, this finding matches previous trends seen for *Wolbachia* strains found as single infections within ants, trends seen among the lepidopteran associates identified here and elsewhere, and broader patterns of host-symbiont specialization that appear to characterize symbiotic associations in general [9], [25], [26], [47], [56].

An important exception to this pattern involves a clade of *wsp* alleles found commonly in ants and lepidopterans. Since many Old World ants harbor *Wolbachia* strains with *wsp* alleles from this clade, it stands to reason that their *Wolbachia* symbionts may have opportunities for genetic exchange amongst a wider gene pool. Whether this does happen, or whether any melting pot effects of multiple infections are more insular, is a question awaiting further investigation.

## Conclusions

The frequencies of heritable symbionts vary across arthropod taxa. *Wolbachia* are the dominant symbionts within several groups, including ants and butterflies, suggesting that this bacterium has likely had a relatively large impact on their ecology and evolution. Correlates of symbiont prevalence provide several clues regarding forces that shape their distributions, identifying promising areas for future research on these pervasive and influential microbes.

## Supporting Information

Checklist S1 **PRISMA 2009 Checklist.**
(DOC)

Figure S1 **Maximum likelihood phylogeny of ant-associated bacteria, their relatives, and heritable symbionts of arthropods.** This full phylogeny is the counterpart to Figure 3 and illustrates relatedness among nearly all known lineages of heritable bacterial symbionts from arthropods, representative sequences from each ant-bacterial 16S rRNA library (i.e. one per 97% phylotype) generated with universal primers, *Arsenophonus* sequences identified with enteric-specific primers, top SeqMatch hits of all representative sequences from ants, and sequences from bacteria previously detected in ants. Symbionts are labeled with their names, and those from larger clades are enclosed by brackets colored the same as the corresponding clade name. Bacterial taxonomy is indicated in the inner color strip. Ant-association (maroon or red) is indicated with branch coloration and with the middle color strip. Symbiont lifestyle (heritable vs. free-living/non-heritable) is indicated with the outer color strip. See second page for figure legend. Notice the large fraction of ant-associated bacteria that do not group with heritable symbionts.
(EPS)

Figure S2 **16S rRNA maximum likelihood phylogeny of other Gammaproteobacteria.** Bootstrap values exceeding 60% are indicated near their respective nodes. Sequences highlighted in red are from ants. Those generated from ants in this study are indicated with “*”. Included in this analysis, in addition to ant-associates, were known members of the larger symbiotic clade including *Sodalis, Buchnera*, *Blochmannia*, etc. along with top BLASTn hits and members of related clades from throughout the enteric bacteria. Note that unlike the other phylogenies, this topology (along with bootstrap values) was generated using RaXML.
(TIFF)

Figure S3 **16S rRNA maximum likelihood phylogeny of** ***Blochmannia*** **and relatives.** Bootstrap values exceeding 60% are indicated near their respective nodes. Sequences generated from ants in this study are indicated with “*”. Those highlighted in red are from ants. Subclades of *Blochmannia* are indicated with labels. Included here, in addition to nearly all known *Blochmannia* sequences, are close relatives identified through BLASTn searches—all are symbionts of other ants.
(EPS)

Figure S4 **16S rRNA maximum likelihood phylogeny of** ***Arsenophonus*** **.** Bootstrap values exceeding 60% are indicated near their respective nodes. Sequences generated from ants in this study are indicated with “*”. Those highlighted in red are from ants, and those in blue are from lepidopterans. Included in this analysis, in addition to ant-associates, were nearly all known *Arsenophonus* sequenes >700 bp in length and representative sequences from clades known to be closely related to *Arsenophonus*.
(EPS)

Information S1 **Details on molecular work, including PCR reactions, purification, cloning, and sequencing, can be found in this section.** Also described are methods for sequence alignment and phylogenetics. This section additionally includes detailed results of taxon-specific phylogenetic analyses and a discussion of the phylogenetic and lifestyle attributes correlating with symbiont distributions.
(DOCX)

Table S1 **PCR conditions used in this study.** *See separate file*.
(DOCX)

Table S2 **PCR and sequencing primer information.** *See separate file*.
(DOCX)

Table S3 **Detailed results of diagnostic PCR screening new to this study.** “+”positive; “−” negative; “?” ambiguous. Screens for *Arsenophonus* involved two primer pairs. Pair 1 (Ars2F & 1513R) results are indicated to the left of the slash and pair 2 (Ars23S-1 & Ars23S-2) results to the right. Note that “n” indicates that one of the two aforementioned pairs was not used. All *Spiroplasma* screen results followed by a “*” were performed with primer pair 63F & TKSSsp; the remaining *Spiroplasma* results were inferred based on BLASTn/phylogenetic results of sequences generated with primer pair cute493F & 1513R. *See Excel file*.
(XLSX)

Table S4 **Intraspecific screening performed for this study.** Numbers of infected individual ants out of the total number with unambiguous screening results are indicated for three symbionts for which intraspecific screening was carried out. *See Excel file*.
(XLSX)

Table S5 **Compiled screening results from this study and those in the systematic review.** Each surveyed species is listed only once in this file, and results are a compilation for each species. “−” indicates species thus far testing negative in all studies; “+” indicates species with at least one sample testing positive in at least one study. Blank cells indicate no (or ambiguous) screening results in our study or those from our systematic review. Cited references performed a diagnostic PCR survey for at least one of the symbionts mentioned within the same row. *See Excel file*.
(XLSX)

Table S6 **Variation in** ***Wolbachia*** **infection frequencies across well-sampled host genera.** Proportions of infected species and sample sizes are indicated for arthropod genera with n>10 species screened for *Wolbachia*. Colors are used to highlight taxa from the same orders. *See Excel file*.
(XLSX)

Table S7 **Results** **of 16S rRNA sequencing with universal and enteric specific primers.** This table provides information on the ant and butterfly hosts whose bacterial communities were targeted with this sequence-based approach. Details on the methodologies used (e.g. cloning vs. direct sequencing, universal vs. enteric primers), the depth of sampling within clone sequence libraries, and the proportions of sequence reads (or clones) belonging to heritable symbionts are also provided.
(XLSX)

Table S8 ***wsp*** **allele query results.** One representative sequence for each unique *wsp* allele found per host was queried against the *wsp* database on the *Wolbachia* MLST website. Listed in this table is information on host taxonomy and (for ants only) geographic origins for arthropods hosting the strains encoding the relevant *wsp* alleles—those identified here and top hits from the database. Color-coding is used to indicate allelic identity, with a key at the bottom of the table. Due to protection of *wsp* allele metadata for some entries in the database not all top hits could be assigned to a host taxon.
(XLSX)

## References

[1] Moran NA, McCutcheon JP, Nakabachi A (2008) Genomics and evolution of heritable bacterial symbionts. Annual Review of Genetics 42: 165–190. 10.1146/annurev.genet.41.110306.130119 18983256

[2] Charlat S, Hurst GDD, Mercot H (2003) Evolutionary consequences of *Wolbachia* infections. Trends in Genetics 19: 217–223. 12683975 10.1016/S0168-9525(03)00024-6

[3] Engelstadter J, Hurst GDD (2009) The ecology and evolution of microbes that manipulate host reproduction. Annual Review of Ecology Evolution and Systematics 40: 127–149.

[4] Oliver KM, Degnan PH, Burke GR, Moran NA (2009) Facultative symbionts in aphids and the horizontal transfer of ecologically important traits. Annual Review of Entomology 55: 247–266. 10.1146/annurev-ento-112408-085305 19728837

[5] Stouthamer R, Breeuwer JAJ, Hurst GDD (1999) *Wolbachia pipientis*: Microbial manipulator of arthropod reproduction. Annual Review of Microbiology 53: 71–102. 10.1146/annurev.micro.53.1.71 10547686

[6] Werren JH (1997) Biology of *Wolbachia* . Annual Review of Entomology 42: 587–609. 10.1146/annurev.ento.42.1.587 15012323

[7] Feldhaar H (2011) Bacterial symbionts as mediators of ecologically important traits of insect hosts. Ecological Entomology 36: 533–543.

[8] Duron O, Wilkes TE, Hurst GDD (2010) Interspecific transmission of a male-killing bacterium on an ecological timescale. Ecology Letters 13: 1139–1148. 20545734 10.1111/j.1461-0248.2010.01502.x

[9] Haselkorn TS, Markow TA, Moran NA (2009) Multiple introductions of the *Spiroplasma* bacterial endosymbiont into *Drosophila* . Molecular Ecology 18: 1294–1305. 19226322 10.1111/j.1365-294X.2009.04085.x

[10] O'Neill SL, Giordano R, Colbert AME, Karr TL, Robertson HM (1992) 16S ribosomal RNA phylogenetic analysis of the bacterial endosymbionts associated with cytoplasmic incompatibility in insects. Proceedings of the National Academy of Sciences of the United States of America 89: 2699–2702. 1557375 10.1073/pnas.89.7.2699 PMC48729

[11] Schilthuizen M, Stouthamer R (1997) Horizontal transmission of parthenogenesis-inducing microbes in *Trichogramma* wasps. Proceedings of the Royal Society of London Series B-Biological Sciences 264: 361–366. 10.1098/rspb.1997.0052 PMC1688260 9107051

[12] Vavre F, Fleury F, Lepetit D, Fouillet P, Bouletreau M (1999) Phylogenetic evidence for horizontal transmission of *Wolbachia* in host-parasitoid associations. Molecular Biology and Evolution 16: 1711–1723. 10605113 10.1093/oxfordjournals.molbev.a026084

[13] Bandi C, Anderson TJC, Genchi C, Blaxter ML (1998) Phylogeny of *Wolbachia* in filarial nematodes. Proceedings of the Royal Society of London Series B-Biological Sciences 265: 2407–2413. 10.1098/rspb.1998.0591 PMC1689538 9921679

[14] Hilgenboecker K, Hammerstein P, Schlattmann P, Telschow A, Werren JH (2008) How many species are infected with *Wolbachia*? - a statistical analysis of current data. FEMS Microbiology Letters 281: 215–220. 18312577 10.1111/j.1574-6968.2008.01110.x PMC2327208

[15] Rousset F, Bouchon D, Pintureau B, Juchault P, Solignac M (1992) *Wolbachia* endosymbionts responsible for various alterations of sexuality in arthropods. Proceedings of the Royal Society of London Series B-Biological Sciences 250: 91–98. 10.1098/rspb.1992.0135 1361987

[16] Werren JH, Windsor D, Guo LR (1995) Distribution of *Wolbachia* among neotropical arthropods. Proceedings of the Royal Society of London Series B-Biological Sciences 262: 197–204.

[17] Duron O, Bouchon D, Boutin S, Bellamy L, Zhou LQ, et al (2008) The diversity of reproductive parasites among arthropods: *Wolbachia* do not walk alone. Bmc Biology 6 10.1186/1741-7007-6-27 PMC2492848 18577218

[18] Perlman SJ, Hunter MS, Zchori-Fein E (2006) The emerging diversity of *Rickettsia* . Proceedings of the Royal Society B-Biological Sciences 273: 2097–2106. 10.1098/rspb.2006.3541 PMC1635513 16901827

[19] Weeks AR, Velten R, Stouthamer R (2003) Incidence of a new sex-ratio-distorting endosymbiotic bacterium among arthropods. Proceedings of the Royal Society of London Series B-Biological Sciences 270: 1857–1865. 10.1098/rspb.2003.2425 PMC1691448 12964989

[20] Weinert LA, Werren JH, Aebi A, Stone GN, Jiggins FM (2009) Evolution and diversity of Rickettsia bacteria. BMC Biology 7 10.1186/1741-7007-7-6 PMC2662801 19187530

[21] Zchori-Fein E, Perlman SJ (2004) Distribution of the bacterial symbiont *Cardinium* in arthropods. Molecular Ecology 13: 2009–2016. 15189221 10.1111/j.1365-294X.2004.02203.x

[22] Baldo L, Prendini L, Corthals A, Werren JH (2007) *Wolbachia* are present in Southern African scorpions and cluster with supergroup F. Current Microbiology 55: 367–373. 17676427 10.1007/s00284-007-9009-4

[23] Rokas A, Atkinson RJ, Nieves-Aldrey JL, West SA, Stone GN (2002) The incidence and diversity of *Wolbachia* in gallwasps (Hymenoptera; Cynipidae) on oak. Molecular Ecology 11: 1815–1829. 12207731 10.1046/j.1365-294x.2002.01556.x

[24] West SA, Cook JM, Werren JH, Godfray HCJ (1998) *Wolbachia* in two insect host-parasitoid communities. Molecular Ecology 7: 1457–1465. 9819901 10.1046/j.1365-294x.1998.00467.x

[25] Baldo L, Ayoub NA, Hayashi CY, Russell JA, Stahlhut JK, et al (2008) Insight into the routes of *Wolbachia* invasion: high levels of horizontal transfer in the spider genus *Agelenopsis* revealed by *Wolbachia* strain and mitochondrial DNA diversity. Molecular Ecology 17: 557–569. 18179432 10.1111/j.1365-294X.2007.03608.x

[26] Russell JA, Goldman-Huertas B, Moreau CS, Baldo L, Stahlhut JK, et al (2009) Specialization and geographic isolation among *Wolachia* symbionts from ants and Lycaenid butterflies. Evolution 63: 624–640. 19054050 10.1111/j.1558-5646.2008.00579.x

[27] Mateos M, Castrezana SJ, Nankivell BJ, Estes AM, Markow TA, et al (2006) Heritable endosymbionts of *Drosophila* . Genetics 174: 363–376. 16783009 10.1534/genetics.106.058818 PMC1569794

[28] Russell JA (2012) The ants (Hymenoptera: Formicidae) are unique and enigmatic hosts of prevalent *Wolbachia* (Alphaproteobacteria) symbionts. Myrmecological News 16: 7–23.

[29] Dunning Hotopp JC, Clark ME, Oliveira D, Foster JM, Fischer P, et al (2007) Widespread lateral gene transfer from intracellular bacteria to multicellular eukaryotes. Science 317: 1753–1756. 17761848 10.1126/science.1142490

[30] Russell JA, Latorre A, Sabater-Munoz B, Moya A, Moran NA (2003) Side-stepping secondary symbionts: widespread horizontal transfer across and beyond the Aphidoidea. Molecular Ecology 12: 1061–1075. 12753224 10.1046/j.1365-294x.2003.01780.x

[31] Zchori-Fein E, Brown JK (2002) Diversity of prokaryotes associated with *Bemisia tabaci* (Gennadius) (Hemiptera : Aleyrodidae). Annals of the Entomological Society of America 95: 711–718.

[32] Hansen AK, Jeong G, Paine TD, Stouthamer R (2007) Frequency of secondary symbiont infection in an invasive psyllid relates to parasitism pressure on a geographic scale in California. Applied and Environmental Microbiology 73: 7531–7535. 17933921 10.1128/AEM.01672-07 PMC2168064

[33] Hedges LM, Brownlie JC, O'Neill SL, Johnson KN (2008) *Wolbachia* and virus protection in insects. Science 322: 702–702. 18974344 10.1126/science.1162418

[34] Jaenike J, Unckless R, Cockburn SN, Boelio LM, Perlman SJ (2010) Adaptation via symbiosis: Recent spread of a *Drosophila* defensive symbiont. Science 329: 212–215. 20616278 10.1126/science.1188235

[35] Oliver KM, Russell JA, Moran NA, Hunter MS (2003) Facultative bacterial symbionts in aphids confer resistance to parasitic wasps. Proceedings of the National Academy of Sciences of the United States of America 100: 1803–1807. 12563031 10.1073/pnas.0335320100 PMC149914

[36] Teixeira L, Ferreira A, Ashburner M (2008) The bacterial symbiont *Wolbachia* induces resistance to RNA viral infections in *Drosophila melanogaster* . Plos Biology 6: 2753–2763. 10.1371/journal.pbio.1000002 PMC2605931 19222304

[37] Moreau CS, Bell CD, Vila R, Archibald SB, Pierce NE (2006) Phylogeny of the ants: Diversification in the age of angiosperms. Science 312: 101–104. 16601190 10.1126/science.1124891

[38] Moreau CS (2009) Inferring ant evolution in the age of molecular data (Hymenoptera: Formicidae). Myrmecological News 12: 201–210.

[39] Cole JR, Chai B, Farris RJ, Wang Q, Kulam SA, et al (2005) The Ribosomal Database Project (RDP-II): sequences and tools for high-throughput rRNA analysis. Nucleic Acids Research 33: D294–D296. 15608200 10.1093/nar/gki038 PMC539992

[40] Miller M, Holder M, Vos R, Midford P, Liebowitz T, et al.. (2011) The CIPRES Portals.

[41] Zwickl D (2006) Genetic algorithm approaches for the phylogenetic analysis of large biological sequence datasets under the maximum likelihood criterion: University of Texas, Austin.

[42] Stamatakis A (2006) RAxML-VI-HPC: Maximum likelihood-based phylogenetic analyses with thousands of taxa and mixed models. Bioinformatics 22: 2688–2690. 16928733 10.1093/bioinformatics/btl446

[43] Letunic I, Bork P (2007) Interactive Tree Of Life (iTOL): an online tool for phylogenetic tree display and annotation. Bioinformatics 23: 127–128. 17050570 10.1093/bioinformatics/btl529

[44] Russell JA, Moreau CS, Goldman-Huertas B, Fujiwara M, Lohman DJ, et al (2009) Bacterial gut symbionts are tightly linked with the evolution of herbivory in ants. Proceedings of the National Academy of Sciences of the United States of America 106: 21236–21241. 19948964 10.1073/pnas.0907926106 PMC2785723

[45] Novakova E, Hypsa V (2007) A new *Sodalis* lineage from bloodsucking fly *Craterina melbae* (Diptera, Hippoboscoidea) originated independently of the tsetse flies symbiont *Sodalis glossinidius* . FEMS Microbiology Letters 269: 131–135. 17227456 10.1111/j.1574-6968.2006.00620.x

[46] Anderson KE, Russell JA, Moreau CS, Kautz S, Sullam KE, et al (2012) Highly similar microbial communities are shared among related and trophically similar ant species. Molecular Ecology 21: 2282–2296. 22276952 10.1111/j.1365-294X.2011.05464.x

[47] Funaro CF, Kronauer DJC, Moreau CS, Goldman-Huertas B, Pierce NE, et al (2011) Army ants harbor a host-specific clade of Entomoplasmatales bacteria. Applied and Environmental Microbiology 77: 346–350. 21075876 10.1128/AEM.01896-10 PMC3019723

[48] Maddison WP, Maddison DR (2003) MacClade: Analysis of phylogeny and character evolution. 4.06 ed. Sunderland, MA: Sinauer Associates.

[49] Swofford D (2001) PAUP*. Sunderland, MA: Sinauer.

[50] Kittayapong P, Jamnongluk W, Thipaksorn A, Milne JR, Sindhusake C (2003) *Wolbachia* infection complexity among insects in the tropical rice-field community. Molecular Ecology 12: 1049–1060. 12753223 10.1046/j.1365-294x.2003.01793.x

[51] Wenseleers T, Ito F, Van Borm S, Huybrechts R, Volckaert F, et al (1998) Widespread occurrence of the micro-organism *Wolbachia* in ants. Proceedings of the Royal Society of London Series B-Biological Sciences 265: 1447–1452. 10.1098/rspb.1998.0456 PMC1689219 9721689

[52] Grindle N, Tyner JJ, Clay K, Fuqua C (2003) Identification of *Arsenophonus-*type bacteria from the dog tick *Dermacentor variabilis* . Journal of Invertebrate Pathology 83: 264–266. 12877836 10.1016/s0022-2011(03)00080-6

[53] Sirvio A, Pamilo P (2010) Multiple endosymbionts in populations of the ant *Formica cinerea* . BMC Evolutionary Biology 10 10.1186/1471-2148-10-335 PMC3087548 21040533

[54] He H, Chen YY, Zhang YL, Wei C (2011) Bacteria associated with gut lumen of *Camponotus japonicus* Mayr. Environmental Entomology 40: 1405–1409. 22217755 10.1603/EN11157

[55] Tufts DM, Bextine B (2009) Identification of bacterial species in the hemolymph of queen *Solenopsis invicta* (Hymenoptera: Formicidae). Environmental Entomology 38: 1360–1364. 19825289 10.1603/022.038.0502

[56] Tsutsui ND, Kauppinen SN, Oyafuso AF, Grosberg RK (2003) The distribution and evolutionary history of *Wolbachia* infection in native and introduced populations of the invasive argentine ant (*Linepithema humile*). Molecular Ecology 12: 3057–3068. 14629385 10.1046/j.1365-294x.2003.01979.x

[57] Benemann J (1973) Nitrogen fixation in termites. Science 181: 164–165. 17746626 10.1126/science.181.4095.164

[58] Breznak J, Brill W, Mertins J, Coppel H (1973) Nitrogen fixation in termites. Nature 244: 577–580. 4582514 10.1038/244577a0

[59] Douglas AE (1998) Nutritional interactions in insect-microbial symbioses: Aphids and their symbiotic bacteria *Buchnera* . Annual Review of Entomology 43: 17–37. 10.1146/annurev.ento.43.1.17 15012383

[60] Xie JL, Vilchez I, Mateos M (2010) *Spiroplasma* bacteria enhance survival of *Drosophila hydei* attacked by the parasitic wasp *Leptopilina heterotoma* . Plos One 5 10.1371/journal.pone.0012149 PMC2921349 20730104

[61] Montllor CB, Maxmen A, Purcell AH (2002) Facultative bacterial endosymbionts benefit pea aphids *Acyrthosiphon pisum* under heat stress. Ecological Entomology 27: 189–195.

[62] Russell JA, Moran NA (2006) Costs and benefits of symbiont infection in aphids: variation among symbionts and across temperatures. Proceedings of the Royal Society B-Biological Sciences 273: 603–610. 10.1098/rspb.2005.3348 PMC1560055 16537132

[63] Kikuchi Y, Hayatsu M, Hosokawa T, Nagayama A, Tago K, et al (2012) Symbiont-mediated insecticide resistance. Proceedings of the National Academy of Sciences of the United States of America 109: 8618–8622. 22529384 10.1073/pnas.1200231109 PMC3365206

[64] Engel P, Martinson VG, Moran NA (2012) Functional diversity within the simple gut microbiota of the honey bee. Proceedings of the National Academy of Sciences of the United States of America 109: 11002–11007. 22711827 10.1073/pnas.1202970109 PMC3390884

[65] Woyke T, Teeling H, Ivanova NN, Huntemann M, Richter M, et al (2006) Symbiosis insights through metagenomic analysis of a microbial consortium. Nature 443: 950–955. 16980956 10.1038/nature05192

[66] Broderick NA, Raffa KF, Goodman RM, Handelsman J (2004) Census of the bacterial community of the gypsy moth larval midgut by using culturing and culture-independent methods. Applied and Environmental Microbiology 70: 293–300. 14711655 10.1128/AEM.70.1.293-300.2004 PMC321235

[67] Pinto-Tomas AA, Sittenfeld A, Uribe-Lorio L, Chavarria F, Mora M, et al (2011) Comparison of midgut bacterial diversity in yropical caterpillars (Lepidoptera: Saturniidae) fed on different diets. Environmental Entomology 40: 1111–1122. 22251723 10.1603/EN11083

[68] Zaspel JM, Hoy MA (2008) Microbial diversity associated with the fruit-piercing and blood-feeding moth *Calyptra thalictri* (Lepidoptera: Noctuidae). Annals of the Entomological Society of America 101: 1050–1055.

[69] Li HW, Medina F, Vinson SB, Coates CJ (2005) Isolation, characterization, and molecular identification of bacteria from the red imported fire ant (*Solenopsis invicta*) midgut. Journal of Invertebrate Pathology 89: 203–209. 16039667 10.1016/j.jip.2005.05.008

[70] Stoll S, Gadau J, Gross R, Feldhaar H (2007) Bacterial microbiota associated with ants of the genus *Tetraponera* . Biological Journal of the Linnean Society 90: 399–412.

[71] van Borm S, Buschinger A, Boomsma JJ, Billen J (2002) *Tetraponera* ants have gut symbionts related to nitrogen-fixing root-nodule bacteria. Proceedings of the Royal Society of London Series B-Biological Sciences 269: 2023–2027. 10.1098/rspb.2002.2101 PMC1691126 12396501

[72] Cafaro MJ, Currie CR (2005) Phylogenetic analysis of mutualistic filamentous bacteria associated with fungus-growing ants. Canadian Journal of Microbiology 51: 441–446. 16121221 10.1139/w05-023

[73] Mueller UG, Dash D, Rabeling C, Rodrigues A (2008) Coevolution between attine ants and actinomycete bacteria: a reevaluation. Evolution 62: 2894–2912. 18752608 10.1111/j.1558-5646.2008.00501.x

[74] Medina F, Li HW, Vinson SB, Coates CJ (2009) Genetic transformation of midgut bacteria from the red imported fire ant (*Solenopsis invicta*). Current Microbiology 58: 478–482. 19159973 10.1007/s00284-008-9350-2

[75] Ishak HD, Plowes R, Sen R, Kellner K, Meyer E, et al (2011) Bacterial diversity in *Solenopsis invicta* and *Solenopsis geminata* ant colonies characterized by 16S amplicon 454 pyrosequencing. Microbial Ecology 61: 821–831. 21243351 10.1007/s00248-010-9793-4

[76] Novakova E, Hypsa V, Moran NA (2009) *Arsenophonus*, an emerging clade of intracellular symbionts with a broad host distribution. BMC Microbiology 9 10.1186/1471-2180-9-143 PMC2724383 19619300

[77] Sebastien A, Gruber MAM, Lester PJ (2012) Prevalence and genetic diversity of three bacterial endosymbionts (*Wolbachia*, *Arsenophonus*, and Rhizobiales) associated with the invasive yellow crazy ant (*Anoplolepis gracilipes*). Insectes Sociaux 59: 33–40.

[78] Shoemaker DD, Ross KG, Keller L, Vargo EL, Werren JH (2000) *Wolbachia* infections in native and introduced populations of fire ants (*Solenopsis* spp.). Insect Molecular Biology 9: 661–673. 11122476 10.1046/j.1365-2583.2000.00233.x

[79] Hurst GDD, Jiggins FM, von der Schulenburg JHG, Bertrand D, West SA, et al (1999) Male-killing *Wolbachia* in two species of insect. Proceedings of the Royal Society of London Series B-Biological Sciences 266: 735–740.

[80] Jiggins FM, Hurst GDD, Jiggins CD, Von der Schulenburg JHG, Majerus MEN (2000) The butterfly *Danaus chrysippus* is infected by a male-killing *Spiroplasma* bacterium. Parasitology 120: 439–446. 10840973 10.1017/s0031182099005867

[81] Bressan A, Terlizzi F, Credi R (2012) Independent origins of vectored plant pathogenic bacteria from arthropod-associated *Arsenophonus* endosymbionts. Microbial Ecology 63: 628–638. 21892672 10.1007/s00248-011-9933-5

[82] Hiroki M, Kato Y, Kamito T, Miura K (2002) Feminization of genetic males by a symbiotic bacterium in a butterfly, Eurema hecabe (Lepidoptera : Pieridae). Naturwissenschaften 89: 167–170. 12061400 10.1007/s00114-002-0303-5

[83] Currie CR, Scott JA, Summerbell RC, Malloch D (1999) Fungus-growing ants use antibiotic-producing bacteria to control garden parasites. Nature 398: 701–704.

[84] Degnan PH, Lazarus AB, Wernegreen JJ (2005) Genome sequence of *Blochmannia pennsylvanicus* indicates parallel evolutionary trends among bacterial mutualists of insects. Genome Research 15: 1023–1033. 16077009 10.1101/gr.3771305 PMC1182215

[85] Feldhaar H, Straka J, Krischke M, Berthold K, Stoll S, et al (2007) Nutritional upgrading for omnivorous carpenter ants by the endosymbiont *Blochmannia* . BMC Biology 5 10.1186/1741-7007-5-48 PMC2206011 17971224

[86] Sauer C, Stackebrandt E, Gadau J, Holldobler B, Gross R (2000) Systematic relationships and cospeciation of bacterial endosymbionts and their carpenter ant host species: proposal of the new taxon *Candidatus* Blochmannia gen. nov. International Journal of Systematic and Evolutionary Microbiology 50: 1877–1886. 11034499 10.1099/00207713-50-5-1877

[87] Jiggins FM, Bentley JK, Majerus MEN, Hurst GDD (2001) How many species are infected with *Wolbachia*? Cryptic sex ratio distorters revealed to be common by intensive sampling. Proceedings of the Royal Society B-Biological Sciences 268: 1123–1126. 10.1098/rspb.2001.1632 PMC1088716 11375098

[88] Sasaki T, Ishikawa H (1999) *Wolbachia* infections and cytoplasmic incompatibility in the almond moth and the mediterranean flour moth. Zoological Science 16: 739–744. 10.2108/zsj.20.153 12655178

[89] van Borm S, Wenseleers T, Billen J, Boomsma JJ (2001) *Wolbachia* in leafcutter ants: a widespread symbiont that may induce male killing or incompatible matings. Journal of Evolutionary Biology 14: 805–814.

[90] Wenseleers T, Sundstrom L, Billen J (2002) Deleterious *Wolbachia* in the ant *Formica truncorum* . Proceedings of the Royal Society of London Series B-Biological Sciences 269: 623–629. 10.1098/rspb.2001.1927 PMC1690935 11916479

[91] Bouwma AM, Shoemaker D (2011) *Wolbachia* wSinvictaA infections in natural populations of the fire ant *Solenopsis invicta*: Testing for phenotypic effects. Journal of Insect Science 11 10.1673/031.011.0111 PMC3281330 21526927

[92] Duron O, Hurst GDD, Hornett EA, Josling JA, Engelstadter J (2008) High incidence of the maternally inherited bacterium *Cardinium* in spiders. Molecular Ecology 17: 1427–1437. 18266629 10.1111/j.1365-294X.2008.03689.x

[93] Martin OY, Goodacre SL (2009) Widespread infections by the bacterial endosymbiont *Cardinium* in arachnids. Journal of Arachnology 37: 106–108.

[94] Perlman SJ, Magnus SA, Copley CR (2010) Pervasive associations between *Cybaeus* spiders and the bacterial symbiont *Cardinium* . Journal of Invertebrate Pathology 103: 150–155. 20035767 10.1016/j.jip.2009.12.009

[95] Jones RT, Bressan A, Greenwell AM, Fierer N (2011) Bacterial communities of two parthenogenetic aphid species cocolonizing two host plants across the Hawaiian islands. Applied and Environmental Microbiology 77: 8345–8349. 21965398 10.1128/AEM.05974-11 PMC3233044

[96] Zchori-Fein E, Gottlieb Y, Kelly SE, Brown JK, Wilson JM, et al (2001) A newly discovered bacterium associated with parthenogenesis and a change in host selection behavior in parasitoid wasps. Proceedings of the National Academy of Sciences of the United States of America 98: 12555–12560. 11592990 10.1073/pnas.221467498 PMC60092

[97] Czarnetzki AB, Tebbe CC (2004) Diversity of bacteria associated with Collembola - a cultivation-independent survey based on PCR-amplified 16S rRNA genes. FEMS Microbiology Ecology 49: 217–227. 19712416 10.1016/j.femsec.2004.03.007

[98] Hail D, Lauziere I, Dowd SE, Bextine B (2011) Culture independent survey of the microbiota of the glassy-winged sharpshooter (*Homalodisca vitripennis*) using 454 pyrosequencing. Environmental Entomology 40: 23–29. 22182607 10.1603/EN10115

[99] Keller L, Liautard C, Reuter M, Brown WD, Sundstrom L, et al (2001) Sex ratio and Wolbachia infection in the ant Formica exsecta. Heredity 87: 227–233. 11703514 10.1046/j.1365-2540.2001.00918.x

[100] Dedeine F, Ahrens M, Calcaterra L, Shoemaker DD (2005) Social parasitism in fire ants (*Solenopsis* spp.): a potential mechanism for interspecies transfer of *Wolbachia* . Molecular Ecology 14: 1543–1548. 15813792 10.1111/j.1365-294X.2005.02499.x

[101] Reuter M, Keller L (2003) High levels of multiple Wolbachia infection and recombination in the ant Formica exsecta. Molecular Biology and Evolution 20: 748–753. 12679529 10.1093/molbev/msg082

[102] Riegler M, Stauffer C (2002) Wolbachia infections and superinfections in cytoplasmically incompatible populations of the European cherry fruit fly Rhagoletis cerasi (Diptera, Tephritidae). Molecular Ecology 11: 2425–2434. 12406252 10.1046/j.1365-294x.2002.01614.x

[103] Kyei-Poku GK, Colwell DD, Coghlin P, Benkel B, Floate KD (2005) On the ubiquity and phylogeny of *Wolbachia* in lice. Molecular Ecology 14: 285–294. 15643971 10.1111/j.1365-294X.2004.02409.x

