# Supplementary material for: A Veritable Menagerie of Heritable Bacteria from Ants, Butterflies, and Beyond: Broad Molecular Surveys and a Systematic Review
Source: PLoS One. 2012 Dec 20;7(12):e51027. doi: 10.1371/journal.pone.0051027 (PMC3527441; doi:10.1371/journal.pone.0051027)
Supplement: Information S1 — Details on molecular work, including PCR reactions, purification, cloning, and sequencing, can be found in this section. Also described are methods for sequence alignment and phylogenetics. This section additionally includes detailed results of taxon-specific phylogenetic analyses and a discussion of the phylogenetic and lifestyle attributes correlating with symbiont distributions. (DOCX) [file pone.0051027.s006.docx]

**Supplementary Methods**

*General methodologies—DNA extractions*

DNA was extracted from ethanol-preserved insects, using the DNeasy Tissue Kit (Qiagen Inc., Valencia, CA) according to the manufacturer’s protocol. Nearly all ant extractions were performed on single workers, after rinsing in sterile water and ethanol, with immatures and males being included for only those species targeted for intraspecific screening. For lepidopterans, extractions targeted a variety of tissues, including legs and abdominal segments. The quality of all novel lepidopteran and ant extractions was assessed by amplifying part of the insect *COI* gene with the primers Ben and Jerry [[1](#_ENREF_1)], as reported in a prior study [[2](#_ENREF_2)].

*General methodologies—PCR*

PCR reactions for diagnostic screening and universal 16S rRNA amplification typically consisted of the following cycling conditions: 1) 94˚C for 2 minutes; 35 cycles of 2) 94˚C for 1 minute, 3) 55-57˚C for 1 minute, and 4) 72°C for 1-2 minutes; and 5) an extension step of 72˚C for 10 minutes. The PCR cocktail recipe for 10 µl screening reactions was: 4.92-5.32 µl H_2_O, 1 µl Qiagen 10X Taq polymerase buffer (with Mg^++^ at 15 mM), 1 µl dNTP mix (25 mM per nucleotide), 0.2-1.0 µl Mg^++^ at 25 mM, 0.8 µl of each primer (5 µM), 0.08 µl of Qiagen Taq polymerase (5 units/µl), and 0.4 µl of DNA template (or water for negative controls). Reactions were scaled up to 25 µl for cloning and direct sequencing. Specific recipes and cycling programs are provided in Table S1, while information on PCR primers is presented in Table S2.

Insects infected with the targeted symbionts were used as positive controls for all screening reactions. DNA extracted from *Triatoma infestans* and *Encarsia pergandiella* served as positive controls for *Arsenophonus* and *Cardinium*, respectively. *Acyrthosiphon pisum* DNA was used as a positive control for *Hamiltonella*. Genomic DNA samples from various ants that had previously screened positive for *Wolbachia* or *Spiroplasma* were used as positive controls in assays for these symbionts. Although not a focus of our study, we also performed some screening for the primary symbiont *Blochmannia*, using *Camponotus pennsylvanicus* as a positive control. To rule out the possibility of contamination, we included a negative control of water in each PCR reaction (in place of DNA).

*General methodologies—PCR purification, cloning, and sequencing*

All PCR products were run on 1% agarose gels and visualized under UV light. Prior to cloning or sequencing, PCR products were purified using an Exo-Ap protocol. Briefly, templates yielding PCR positives were re-amplified in 25 µl reactions and purified by adding 1 µl of Antarctic phosphatase, 1 µl of Antarctic phosphatase buffer, and 0.6 µl of *E. coli* exonuclease I (New England Biolabs, Ipswich, MA). Clean-up reactions were incubated in a thermocycler at 37˚C for 35 minutes, followed by 80˚C for 20 minutes.

For cloning, purified PCR products (see below) were ligated into the pCR2.1 vector from Invitrogen. Ligation products were then used to transform One Shot chemically competent *E. coli* cells (Invitrogen, Carlsbad, CA). Blue-white colony screening on LB plates with Kanamycin allowed us to identify transformants with successful ligation. After picking and growing white colonies overnight in LB broth, we extracted DNA from each liquid culture by boiling for 10 minutes. Extracted DNA was used for insert-size assays with primers M13F and M13R. Amplified products of the expected size were subsequently purified for DNA sequencing as described below.

Our sequencing studies typically targeted two genes—the 16S rRNA gene of bacteria and the *wsp* gene of *Wolbachia*. While all new16S rRNA sequences and several *wsp* sequences were generated by Macrogen, USA, most *wsp* amplicons were sequenced at Harvard University. In these instances, products were generated using the ABI PRISM^®^ BigDye Terminator Cycle Sequencing Kit v.3.1 (Applied Biosystems) and run on an ABI PRISM^®^ 3100 Genetic Analyzer. Sequence fragments were subsequently edited and assembled using Sequencher version 4.2 [[3](#_ENREF_3)].

*Diagnostic PCR assays*

Screening for *Wolbachia* utilized *wsp* primers wsp81F and wsp691R [[4](#_ENREF_4)]. New *Wolbachia* screening across ants was limited to members of the tribe Camponotini from the genus *Polyrhachis* (n=24 species) and army ants from the subfamilies Aenictinae, Dorylinae, and Ecitoninae (n=56 species). New surveys across the Lepidoptera were more widespread, targeting 165 species from all 13 aforementioned families. All *Wolbachia* infections in lepidopterans and army ants were confirmed by sequencing of *wsp*, while frequent instances of low-quality *wsp* sequences for some camponotines often necessitated *Wolbachia* confirmation through sequencing of various housekeeping genes [[5](#_ENREF_5)].

Surveys for the symbionts *Arsenophonus*, *Cardinium hertigii*, *Hamiltonella defensa*, and *Spiroplasma* spanned a broader range of species, as reported in the Results section and Table S3. *H. defensa* screens were employed with the diagnostic primers T1279F and 35R as described previously [[6](#_ENREF_6)].

PCR assays for *Arsenophonus* and *Cardinium* utilized newly designed diagnostic primers. To design primers, we downloaded 16S rRNA sequences from bacteria in these two clades and from closely related bacteria, which we did not wish to target in our screening assays. After sequence alignment, we scanned the resulting matrices for regions that were conserved within the *Arsenophonus* and *Cardinium* clades, while differing at the 3’ end from 16S rRNA sequences of outgroup bacteria. For both symbionts we ordered several candidate diagnostic primers, utilizing these in combination with each other and with universal primers to optimize amplification of 16S rRNA genes from positive control templates. Of the selected PCR assays, all were predicted to exhibit good specificity and sensitivity according to ProbeMatch analyses [[7](#_ENREF_7)], comparing favorably with those used in prior publications (data not shown).

In short, *Cardinium* screening employed the use of newly designed diagnostic primers Card211F and Card1310R. *Arsenophonus* screening utilized the newly designed primer Ars2F with universal primer 1513R. A subset of *Arsenophonus* surveys alternatively (or additionally) utilized primers Ars23S-1 with Ars23S-2 [[8](#_ENREF_8)]. Templates amplifying with *Arsenophonus* primers were subsequently rescreened and sequenced with primers F40 and R1060 [[9](#_ENREF_9)], which target enteric bacteria.

Diagnostic *Spiroplasma* screening across 34 ants from the tribe Camponotini used the primer pair 63F and TKSSsp [[10](#_ENREF_10),[11](#_ENREF_11)]. Though not further illustrated here, 33 of these ants were also screened with primers 16STF1 and 16STR1 [[12](#_ENREF_12)]; and in all but one case (a false positive with 16STF1 and 16STR1), the results from this second assay were identical to those of the first. Surveys for *Spiroplasma* across the Lepidoptera utilized the primers cute493F and 1513R [[13](#_ENREF_13)]. Due to the broader range of bacterial taxa that can be detected with this assay, positives were only declared after sequence confirmation and subsequent BLASTn or phylogenetic analyses identified the novel sequences as close relatives of other *Spiroplasma* species. Negatives were declared for templates that did not amplify with this primer pair and for those for which these primers amplified non-*Spiroplasma* bacteria. A similar approach was utilized to estimate *Spiroplasma* frequencies across a broad range of ants studied in Funaro et al. 2011.

Although we designed several diagnostic assays for *Blochmannia* (heritable nutritional symbionts of camponotines), not one was capable of amplifying 16S rRNA genes from all strains, even when these symbionts were subsequently confirmed to be present. However, through a combination of diagnostic primers, we were able to amplify 16S rRNA genes from several novel *Blochmannia* isolates that were used in subsequent phylogenetic analyses. Also, when particular worker ants screened negative in all *Blochmannia*-specific PCR assays, we subsequently targeted these with universal PCR, cloning, and sequencing (described in a subsequent section) to further search for this symbiont.

*Analyzing universal 16S rRNA sequences*

All new 16S rRNA sequences generated with universal primers (from 19 ant species and one lepidopteran) or enteric specific primers (from three ants and one lepidopteran) were checked for chimeras using Bellerophon version 3 [[14](#_ENREF_14)]. Among these, all but fourteen identified chimeric sequences were then separately uploaded to the Ribosomal Database Project (RDP) database [[7](#_ENREF_7)] for sequence alignment. The same was done for previously generated universal sequences from 62 ant species [[13](#_ENREF_13),[15](#_ENREF_15),[16](#_ENREF_16)].

We analyzed a total of 474 universal 16S rRNA sequences sampled from 82 individual ants spanning 78 colonies and 74 species. The number of universal sequence reads per ant host ranged from 1-27, with an average of 5.7 (see Table S4 for sample sizes and for the identity of host species sampled).

All clone library alignments with n>2 reads were uploaded to the program DivergentSet [[17](#_ENREF_17)]. Using this program, one representative sequence was selected from each 97% phylotype for subsequent analyses. Each of these representatives, and all of those from direct universal sequencing, was uploaded to the RDP website where SeqMatch searches were performed to identify top relatives [[7](#_ENREF_7)]. SeqMatch hits were then aligned, along with the universal 16S rRNA sequences from ants and lepidopterans and those from known heritable symbionts spanning the bacterial phylogeny.

After maximum likelihood analyses, clustering with known heritable symbionts on the generated phylogeny was used to suggest the potential for heritability among the novel bacteria identified from ants and lepidopterans.

**Supplementary Results**

*Taxon-specific phylogenetic analyses*

Maximum likelihood phylogenies were constructed for *Arsenophonus*, *Spiroplasma*, *Blochmannia*, and heritable enteric bacteria (Figure 4; Figures S2, S3, S4) to further assess the relationships between ant- and lepidopteran-associated bacteria and known heritable symbionts. Each phylogenetic analysis included ant-associates, top relatives detected through SeqMatch hits, plus representative members of heritable bacterial clades. On the *Arsenophonus* phylogeny (Figure S4), bacteria identified from four ant species grouped into three different lineages. Those from two army ant hosts identified in this study grouped with strains from whiteflies, aphids, and psyllids, albeit with low bootstrap support. Another bacterium identified here, from *Technomyrmex albipes*, was basal to a clade consisting of strains from blood-feeding insect hosts. This strain was also found in a separate lineage than a previously identified strain from this same species, although bootstrap support for its placement was low.

Additionally, one ant host of *Arsenophonus* and three lepidopterans from our study harbored bacteria that clustered into small, insect-associated lineages within the genus *Providencia*. Although detected with diagnostic primers, their phylogenetic placement indicated that they should not be labeled as *Arsenophonus* bacteria.

The *Spiroplasma* phylogeny (Figure 4) revealed that previously described strains from lepidopterans were related to known heritable symbionts, but also to plant pathogens from the citri group and to microbes from horse- and deer-flies. When considering the ant-associates, eight out of ten previously described strains grouped with *Spiroplasma platyhelix*, a dragonfly associate that is not known to undergo vertical transmission. Six newly identified *Polyrhachis*-associated *Spiroplasma* fell into three disparate lineages. One strain grouped into the platyhelix lineage, falling out as the basal branch to this largely ant-associated group. A second strain grouped within the ixodetis clade, falling on a branch that clustered with a heritable male-killing microbe from the butterfly *Danaus chrysippus*. In addition, a total of four strains from *Polyrhachis* grouped within the citri lineage of *Spiroplasma*. These showed relatedness to heritable (non-male-killing) symbionts from four *Drosophila* species and to an ant-associated bacterium with an unknown lifestyle and transmission mode.

In addition to these *Spiroplasma* strains, two bacteria from *Polyrhachis* hosts grouped outside of known *Spiroplasma* clades, clustering instead within a lineage of Entomoplasmataceae. The containing clade was comprised of a *Mesoplasma* species from plants and of microbes from various ant species.

The phylogeny of *Blochmannia* and relatives (Figure S3) recovered the expected monophyly of *Blochmannia* symbionts. Within this lineage, branching patterns were similar to those reported previously by Wernegreen and colleagues [[18](#_ENREF_18)], with microbes from *Camponotus*, the *Colobopsis* subgenus of *Camponotus*, *Polyrhachis*, and *Echinopla*/*Calomyrmex* grouping into separate lineages. Also similar to previous findings was the observation that *Echinopla*/*Calomyrmex* associates clustered with the majority of the *Camponotus* associates from hosts outside the subgenus *Colobopsis*. In their prior study, Wernegreen and colleagues also observed that the closest relatives of *Blochmannia* are secondary symbionts of scale insects. We obtained a similar trend in one of the two phylogenies constructed for this group for which nucleotides from a hard-to-align insertion were removed. When the insertion was included in the alignment, the resulting phylogeny suggested that a bacterium from the ant *Cardiocondyla emeryi* was the closest relative of the *Blochmannia* clade (data not shown).

The Gammaproteobacteria phylogeny (Figure S2) revealed several other notable trends for ant-associated bacteria, implicating several as candidate heritable symbionts based on their phylogenetic affinities. For instance, a bacterium from *Cardiocondyla emeryi* was most closely related to the heritable primary symbiont *Baumannia cicadellinicola* from a leafhopper (*Helochara communis*) and a secondary symbiont of a psyllid (*Aphalaroida inermis*). This lineage, in turn, was related to symbionts from sap-feeding insects, including scale insects, psyllids, leeches, and aphids, including several with demonstrated maternal transmission. Sister to this clade was a group containing heritable *Blochmannia* symbionts and bacteria from the ant *Notostigma carazzii*. Outside of this lineage were gut bacteria from *Tetraponera* ants, and bacteria from the ant genera *Iridomyrmex*, *Euprenolepis*, and *Plagiolepis*. Microbes from these latter three host genera clustered with a *Sodalis* symbiont of tsetse flies, along with symbionts from other insects with nutrient-poor diets, such as scale insects, hippoboscid flies, lice, and grain weevils. Many of the bacteria from this lineage are also known to be heritable, and when looking at the larger clade containing each of the aforementioned bacteria, we see that all are insect-associated symbionts.

Some ant associated bacteria fell outside of this group. For instance, bacteria identified from *Crematogaster navajoa*, a wood ant (*Formica fusca*), and the garden of a leaf-cutter ant (*Atta colombica*) clustered with *Escherichia* and *Enterobacter*. Ant associates also came from clades containing *Pantoea* and *Serratia* species.

**Supplementary Discussion**

*Why are* Wolbachia *more prevalent than other symbionts?*

One possible explanation for high *Wolbachia* prevalence could stem from recombination, as the capacity to shuffle alleles and protein domains across this species [[19-23](#_ENREF_19)] may facilitate adaptation to novel hosts. Indeed, it has been noted that greater genetic variability within a species correlates with greater host range [[24](#_ENREF_24)]. It is not yet known whether most other facultative heritable symbionts undergo substantial recombination (but see [[25](#_ENREF_25)] for recent insights into *Arsenophonus*), although recent studies within *Spiroplasma* and *Hamiltonella* suggest lower recombination rates than those seen for *Wolbachia* [[12](#_ENREF_12),[26](#_ENREF_26)].

The possibility that *Wolbachia* is an older group that has had more time to colonize a broader range of hosts is unlikely for two reasons. First, there is no clear rule about how much time is required for host range expansion; and in fact, 16S rRNA divergence suggests that all lineages targeted here are many tens of millions of years old. Second, genetic divergence levels do not make it clear that the *Wolbachia* lineages that are most widespread across arthropods (Supergroups A and B) are any older than those of rarer, more host-range-restricted bacteria. Assuming an accelerated rate of sequence evolution of 4-8% divergence per 100 million years in the 16S rRNA gene [[27](#_ENREF_27),[28](#_ENREF_28)], one would estimate their divergence time to be very roughly 25-50 million years since the last common ancestor of Supergroup A and B *Wolbachia*, based on their 2% divergence [[29](#_ENREF_29)]. In light of this, it is interesting to note that divergence between the two most distantly related clades of *Cardinium* (i.e. those from the Opiliones (daddy-long-legs) vs. all other known members of this lineage) is over 5% [[30](#_ENREF_30)], while divergence between *Hamiltonella* and its symbiotic sister genus *Regiella* (with an apparently limited host range) is ~8% [[6](#_ENREF_6)]. All else being equal, it does not appear then that *Wolbachia* have had a head start on their less widespread and less common counterparts.

Alternative factors to explain differences in both host range and incidence could relate to symbionts’ abilities to disperse horizontally between hosts, perhaps through the diet, spread by predator or parasitoid vectors, wound-to-wound contact, and/or parasitoid co-infection [[31-33](#_ENREF_31)]. Perhaps the sheer variety of strategies used by *Wolbachia* to persist within host populations has enabled them to proliferate across many different groups. To date, the Swiss Army Knife repertoire employed by these symbionts includes vitamin biosynthesis, iron metabolism, four types of reproductive manipulation, and defense against microbes [[29](#_ENREF_29),[34-37](#_ENREF_34)]. While *Wolbachia* are far better studied than other heritable symbionts, to date no other microbe approaches this variety of life history tactics.

*What attributes correlate with symbiont prevalence?*

One previously reported trend for *Wolbachia* prevalence is a rarity within two host groups engaging in cyclical parthenogenesis—namely the Cynipini (oak gall wasps) and aphids [[38](#_ENREF_38),[39](#_ENREF_39)]. This could extend from a reduced opportunity to manipulate host reproduction, given the partial reliance on asexual reproduction by these insects (which is not mediated by symbionts).

Among ants, prior results suggest that the mode of colony founding is suggestively correlated with *Wolbachia* infection: there are fewer infections among species characterized by obligately independent colony founding in which queens disperse alone (typically through flight) to found new nests [[40](#_ENREF_40),[41](#_ENREF_41)]. Perhaps the high costs of dispersal and unassisted nest initiation make infected hosts more prone to failure in the presence of even slight costs imposed by their symbionts. Yet it should be noted that even dependent-founding taxa show extensive variation in *Wolbachia* infection frequencies, with high levels in the army ant genus *Aenictus* compared to low rates of infection in the related genus *Dorylus*. In these cases, the potential drivers of frequency differences remain unclear. Furthermore, the statistical support for the effect of colony founding mode was previously found to be only marginally significant [[40](#_ENREF_40),[41](#_ENREF_41)], suggesting a need for further investigation.

In considering correlates of infection for other symbionts, *Arsenophonus* appear common among arthropods that derive the majority of their nutrition from blood. This is true for the bat flies, kissing bugs, lice, etc., where highly prevalent *Arsenophonus* infections and (in some cases) cospeciation may actually indicate that these symbionts are required by their hosts [[42-45](#_ENREF_42)]. Enrichment in unrelated insects with similar, nutritionally insufficient diets suggests a nutritional role for these bacteria, and the retention of pathways for vitamin biosynthesis in at least one *Arsenophonus* strain suggests an ability to serve in such a capacity [[46](#_ENREF_46)]. Indeed, it is known that vitamin biosynthesis is a common service provided by symbionts of other blood-feeding arthropods [[36](#_ENREF_36),[47](#_ENREF_47)].

*Arsenophonus* are also enriched among a number of sap-feeding insects. While this could imply a different nutritional role that is specific for this niche, most known nutritional symbionts are fixed within their host species, which does not fit current knowledge of *Arsenophonus* infection in some sap-feeders [[48](#_ENREF_48),[49](#_ENREF_49)]. It is interesting to note that a large percentage of the sap-feeding hosts harboring *Arsenophonus* also harbor obligate, primary symbionts that are involved in their nutrition. So perhaps *Arsenophonus* are well-suited for lifestyles in organisms with the cells, organs, and other mechanisms already in place for the maintenance of bacterial symbionts [[50](#_ENREF_50)]. Or perhaps such hosts are commonly reliant upon defensive bacterial symbionts, a role that has been hypothesized for *Arsenophonus* in psyllids [[51](#_ENREF_51)]. Similar arguments could be made for *Hamiltonella*, a microbe with no hypothesized nutritional roles that is common among sap-feeding insects such as aphids and whiteflies [[6](#_ENREF_6),[52](#_ENREF_52)]. The known defensive capacities of this microbe [[53](#_ENREF_53)] suggest that hosts benefiting from supplemental defense might permit its spread. Should other sap-feeders prove similar to pea aphids, common hosts of defensive symbionts that have lost several host-encoded immune responses [[54](#_ENREF_54),[55](#_ENREF_55)], this would elevate host immunology as a candidate determinant of symbiont distributions.

*Spiroplasma* appear broadly distributed across a heterogeneous range of host lineages with no clear biological similarities. Many strains of *Spiroplasma* are not heritable but are instead associated with insect guts or committed to lifestyles as pathogens [[56](#_ENREF_56)]. In this study, strains that grouped with known heritable *Spiroplasma* symbionts were mostly limited to the Lepidoptera and to *Polyrhachis* ants. Indeed, within this ant genus, *Spiroplasma* were quite common, although strains hailed from multiple lineages. Given the presence of the primary symbiont *Blochmannia* in this group, it is possible that these ants are especially hospitable to secondary symbionts [[50](#_ENREF_50)]. Yet this does not explain why *Spiroplasma* enrichment seemed confined to just one genus of *Blochmannia* hosts. Regardless, the lifestyles, tissue tropism, and transmission modes of *Spiroplasma* clearly must be studied in greater detail before we can better understand their prevalence in *Polyrhachis*.

Aside from these ants, *Spiroplasma* tend to be found at low levels across host taxa [[57](#_ENREF_57)], suggesting that they are more often side-shows than show-stealing stars. Nevertheless, their prevalence within some host species, and their roles as defenders and reproductive manipulators [[58-60](#_ENREF_58)], suggest that these symbionts may still be important for a diverse minority of the world’s arthropod species.

The last of our surveyed bacteria, *Cardinium*, shows enrichment in daddy-long-legs, spiders, biting midges, and planthoppers [[30](#_ENREF_30),[61](#_ENREF_61),[62](#_ENREF_62)]. In addition, *Cardinium* are commonly found in a limited range of families with frequent, and possibly universal, haplodiploidy—the Aphelinidae (parasitic wasps), the Diaspididae (armored scales), and each of the Tenuipalpidae, Tetranychidae, and Phytoseiidae (mites) [[62-65](#_ENREF_62)]. *Cardinium* have similarly been found within haplodiploid whitefly species [[49](#_ENREF_49),[64](#_ENREF_64)]. While host-parasitoid interactions may facilitate horizontal transfer among some of these groups [[63](#_ENREF_63)], haplodiploidy is clearly a thread that ties many *Cardinium* hosts together. This raises questions about cause and effect in this relationship, and whether reproductive manipulation by this symbiont is somehow facilitated in haplodiploid backgrounds. Regardless of the explanation, the rarity of *Cardinium* across some families of haplodiploid hymenopterans provides a clear indication that haplodiploidy alone is not responsible for the patchy range of this heritable symbiont.

**Supplementary References Cited**

1. Simon C, Frati F, Beckenbach A, Crespi B, Liu H, et al. (1994) Evolution, weighting, and phylogenetic utility of mitochondrial gene-sequences and a compilarion of conserved polymerase chain-reaction primers Annals of the Entomological Society of America 87: 651-701.

2. Russell JA, Goldman-Huertas B, Moreau CS, Baldo L, Stahlhut JK, et al. (2009) Specialization and geographic isolation among *Wolachia* symbionts from ants and Lycaenid butterflies. Evolution 63: 624-640.

3. Genecodes (2003) Sequencher. 4.2 ed. Ann Arbor, MI: Genecodes Co.

4. Zhou WG, Rousset F, O'Neill S (1998) Phylogeny and PCR-based classification of Wolbachia strains using wsp gene sequences. Proceedings of the Royal Society of London Series B-Biological Sciences 265: 509-515.

5. Baldo L, Hotopp JCD, Jolley KA, Bordenstein SR, Biber SA, et al. (2006) Multilocus sequence typing system for the endosymbiont Wolbachia pipientis. Applied and Environmental Microbiology 72: 7098-7110.

6. Russell JA, Latorre A, Sabater-Munoz B, Moya A, Moran NA (2003) Side-stepping secondary symbionts: widespread horizontal transfer across and beyond the Aphidoidea. Molecular Ecology 12: 1061-1075.

7. Cole JR, Chai B, Farris RJ, Wang Q, Kulam SA, et al. (2005) The Ribosomal Database Project (RDP-II): sequences and tools for high-throughput rRNA analysis. Nucleic Acids Research 33: D294-D296.

8. Thao MLL, Baumann P (2004) Evidence for multiple acquisition of Arsenophonus by whitefly species (Sternorrhyncha : Aleyrodidae). Current Microbiology 48: 140-144.

9. Novakova E, Hypsa V (2007) A new *Sodalis* lineage from bloodsucking fly *Craterina melbae* (Diptera, Hippoboscoidea) originated independently of the tsetse flies symbiont *Sodalis glossinidius*. FEMS Microbiology Letters 269: 131-135.

10. Fukatsu T, Nikoh N (2000) Endosymbiotic microbiota of the bamboo pseudococcid Antonina crawii (Insecta, Homoptera). Applied and Environmental Microbiology 66: 643-650.

11. Mateos M, Castrezana SJ, Nankivell BJ, Estes AM, Markow TA, et al. (2006) Heritable endosymbionts of *Drosophila*. Genetics 174: 363-376.

12. Haselkorn TS, Markow TA, Moran NA (2009) Multiple introductions of the *Spiroplasma* bacterial endosymbiont into *Drosophila*. Molecular Ecology 18: 1294-1305.

13. Funaro CF, Kronauer DJC, Moreau CS, Goldman-Huertas B, Pierce NE, et al. (2011) Army ants harbor a host-specific clade of Entomoplasmatales bacteria. Applied and Environmental Microbiology 77: 346-350.

14. Huber T, Faulkner G, Hugenholtz P (2004) Bellerophon: a program to detect chimeric sequences in multiple sequence alignments. Bioinformatics 20: 2317-2319.

15. Russell JA, Moreau CS, Goldman-Huertas B, Fujiwara M, Lohman DJ, et al. (2009) Bacterial gut symbionts are tightly linked with the evolution of herbivory in ants. Proceedings of the National Academy of Sciences of the United States of America 106: 21236-21241.

16. Anderson KE, Russell JA, Moreau CS, Kautz S, Sullam KE, et al. (2012) Highly similar microbial communities are shared among related and trophically similar ant species. Molecular Ecology 21: 2282-2296.

17. Widmann J, Hamady M, Knight R (2006) DivergentSet, a tool for picking non-redundant sequences from large sequence collections. Molecular & Cellular Proteomics 5: 1520-1532.

18. Wernegreen JJ, Kauppinen SN, Brady SG, Ward PS (2009) One nutritional symbiosis begat another: Phylogenetic evidence that the ant tribe Camponotini acquired Blochmannia by tending sap-feeding insects. Bmc Evolutionary Biology 9.

19. Baldo L, Bordenstein S, Wernegreen JJ, Werren JH (2006) Widespread recombination throughout *Wolbachia* genomes. Molecular Biology and Evolution 23: 437-449.

20. Baldo L, Lo N, Werren JH (2005) Mosaic nature of the *Wolbachia* surface protein. Journal of Bacteriology 187: 5406-5418.

21. Bordenstein SR, Wernegreen JJ (2004) Bacteriophage flux in endosymbionts (*Wolbachia*): Infection frequency, lateral transfer, and recombination rates. Molecular Biology and Evolution 21: 1981-1991.

22. Jiggins FM, von der Schulenburg JHG, Hurst GDD, Majerus MEN (2001) Recombination confounds interpretations of *Wolbachia* evolution. Proceedings of the Royal Society B-Biological Sciences 268: 1423-1427.

23. Werren JH, Bartos JD (2001) Recombination in *Wolbachia*. Current Biology 11: 431-435.

24. Woolhouse MEJ, Taylor LH, Haydon DT (2001) Population biology of multihost pathogens. Science 292: 1109-1112.

25. Mouton L, Thierry M, Henri H, Baudin R, Gnankine O, et al. (2012) Evidence of diversity and recombination in *Arsenophonus* symbionts of the *Bemisia tabaci* species complex. Bmc Microbiology 12.

26. Degnan PH, Moran NA (2008) Evolutionary genetics of a defensive facultative symbiont of insects: exchange of toxin-encoding bacteriophage. Molecular Ecology 17: 916-929.

27. Moran NA (1996) Accelerated evolution and Muller's rachet in endosymbiotic bacteria. Proceedings of the National Academy of Sciences of the United States of America 93: 2873-2878.

28. Moran NA, Munson MA, Baumann P, Ishikawa H (1993) A molecular clock in endosymbiotic bacteria is calibrated using the insect hosts. Proceedings of the Royal Society of London Series B-Biological Sciences 253: 167-171.

29. Werren JH (1997) Biology of *Wolbachia*. Annual Review of Entomology 42: 587-609.

30. Chang J, Masters A, Avery A, Werren JH (2010) A divergent *Cardinium* found in daddy long-legs (Arachnida: Opiliones). Journal of Invertebrate Pathology 105: 220-227.

31. Duron O, Wilkes TE, Hurst GDD (2010) Interspecific transmission of a male-killing bacterium on an ecological timescale. Ecology Letters 13: 1139-1148.

32. Huigens ME, Luck RF, Klaassen RHG, Maas M, Timmermans M, et al. (2000) Infectious parthenogenesis. Nature 405: 178-179.

33. Jaenike J, Polak M, Fiskin A, Helou M, Minhas M (2007) Interspecific transmission of endosymbiotic *Spiroplasma* by mites. Biology Letters 3: 23-25.

34. Brownlie JC, Cass BN, Riegler M, Witsenburg JJ, Iturbe-Ormaetxe I, et al. (2009) Evidence for metabolic provisioning by a common invertebrate endosymbiont, *Wolbachia pipientis*, during periods of nutritional stress. Plos Pathogens 5.

35. Hedges LM, Brownlie JC, O'Neill SL, Johnson KN (2008) *Wolbachia* and virus protection in insects. Science 322: 702-702.

36. Hosokawa T, Koga R, Kikuchi Y, Meng XY, Fukatsu T (2010) *Wolbachia* as a bacteriocyte-associated nutritional mutualist. Proceedings of the National Academy of Sciences of the United States of America 107: 769-774.

37. Teixeira L, Ferreira A, Ashburner M (2008) The bacterial symbiont *Wolbachia* induces resistance to RNA viral infections in *Drosophila melanogaster*. Plos Biology 6: 2753-2763.

38. Rokas A, Atkinson RJ, Nieves-Aldrey JL, West SA, Stone GN (2002) The incidence and diversity of *Wolbachia* in gallwasps (Hymenoptera; Cynipidae) on oak. Molecular Ecology 11: 1815-1829.

39. West SA, Cook JM, Werren JH, Godfray HCJ (1998) *Wolbachia* in two insect host-parasitoid communities. Molecular Ecology 7: 1457-1465.

40. Russell JA (2012) The ants (Hymenoptera: Formicidae) are unique and enigmatic hosts of prevalent *Wolbachia* (Alphaproteobacteria) symbionts. Myrmecological News 16: 7-23.

41. Wenseleers T, Ito F, Van Borm S, Huybrechts R, Volckaert F, et al. (1998) Widespread occurrence of the micro-organism *Wolbachia* in ants. Proceedings of the Royal Society of London Series B-Biological Sciences 265: 1447-1452.

42. Allen JM, Reed DL, Perotti MA, Braig HR (2007) Evolutionary relationships of "Candidatus *Riesia* spp.," endosymbiotic Enterobacteriaceae living within hematophagous primate lice. Applied and Environmental Microbiology 73: 1659-1664.

43. Hosokawa T, Nikoh N, Koga R, Sato M, Tanahashi M, et al. (2012) Reductive genome evolution, host-symbiont co-speciation and uterine transmission of endosymbiotic bacteria in bat flies. Isme Journal 6: 577-587.

44. Novakova E, Hypsa V, Moran NA (2009) *Arsenophonus*, an emerging clade of intracellular symbionts with a broad host distribution. BMC Microbiology 9.

45. Sorfova P, Skerikova A, Hypsa V (2008) An effect of 16S rRNA intercistronic variability on coevolutionary analysis in symbiotic bacteria: Molecular phylogeny of *Arsenophonus triatominarum*. Systematic and Applied Microbiology 31: 88-100.

46. Darby AC, Choi JH, Wilkes T, Hughes MA, Werren JH, et al. (2010) Characteristics of the genome of Arsenophonus nasoniae, son-killer bacterium of the wasp Nasonia. Insect Molecular Biology 19: 75-89.

47. Akman L, Yamashita A, Watanabe H, Oshima K, Shiba T, et al. (2002) Genome sequence of the endocellular obligate symbiont of tsetse flies, *Wigglesworthia glossinidia*. Nature Genetics 32: 402-407.

48. Jones RT, Bressan A, Greenwell AM, Fierer N (2011) Bacterial communities of two parthenogenetic aphid species cocolonizing two host plants across the Hawaiian islands. Applied and Environmental Microbiology 77: 8345-8349.

49. Skaljac M, Zanic K, Ban SG, Kontsedalov S, Ghanim M (2010) Co-infection and localization of secondary symbionts in two whitefly species. BMC Microbiology 10.

50. Koga R, Meng XY, Tsuchida T, Fukatsu T (2012) Cellular mechanism for selective vertical transmission of an obligate insect symbiont at the bacteriocyte-embryo interface. Proceedings of the National Academy of Sciences of the United States of America 109: E1230-E1237.

51. Hansen AK, Jeong G, Paine TD, Stouthamer R (2007) Frequency of secondary symbiont infection in an invasive psyllid relates to parasitism pressure on a geographic scale in California. Applied and Environmental Microbiology 73: 7531-7535.

52. Zchori-Fein E, Brown JK (2002) Diversity of prokaryotes associated with *Bemisia tabaci* (Gennadius) (Hemiptera : Aleyrodidae). Annals of the Entomological Society of America 95: 711-718.

53. Oliver KM, Russell JA, Moran NA, Hunter MS (2003) Facultative bacterial symbionts in aphids confer resistance to parasitic wasps. Proceedings of the National Academy of Sciences of the United States of America 100: 1803-1807.

54. Gerardo NM, Altincicek B, Anselme C, Atamian H, Barribeau SM, et al. (2010) Immunity and other defenses in pea aphids, *Acyrthosiphon pisum*. Genome Biology 11.

55. Laughton AM, Garcia JR, Altincicek B, Strand MR, Gerardo NM (2011) Characterisation of immune responses in the pea aphid, *Acyrthosiphon pisum*. Journal of Insect Physiology 57: 830-839.

56. Gasparich GE (2010) Spiroplasmas and phytoplasmas: Microbes associated with plant hosts. Biologicals 38: 193-203.

57. Duron O, Bouchon D, Boutin S, Bellamy L, Zhou LQ, et al. (2008) The diversity of reproductive parasites among arthropods: *Wolbachia* do not walk alone. Bmc Biology 6.

58. Jaenike J, Unckless R, Cockburn SN, Boelio LM, Perlman SJ (2010) Adaptation via symbiosis: Recent spread of a *Drosophila* defensive symbiont. Science 329: 212-215.

59. Jiggins FM, Hurst GDD, Jiggins CD, Von der Schulenburg JHG, Majerus MEN (2000) The butterfly *Danaus chrysippus* is infected by a male-killing *Spiroplasma* bacterium. Parasitology 120: 439-446.

60. Xie JL, Vilchez I, Mateos M (2010) *Spiroplasma* bacteria enhance survival of *Drosophila hydei* attacked by the parasitic wasp *Leptopilina heterotoma*. Plos One 5.

61. Duron O, Hurst GDD, Hornett EA, Josling JA, Engelstadter J (2008) High incidence of the maternally inherited bacterium *Cardinium* in spiders. Molecular Ecology 17: 1427-1437.

62. Nakamura Y, Kawai S, Yukuhiro F, Ito S, Gotoh T, et al. (2009) Prevalence of *Cardinium* bacteria in planthoppers and spider mites and taxonomic revision of "*Candidatus* Cardinium hertigii" based on detection of a new *Cardinium* group from biting midges. Applied and Environmental Microbiology 75: 6757-6763.

63. Gruwell ME, Wu J, Normark BB (2009) Diversity and phylogeny of *Cardinium* (Bacteroidetes) in armored scale insects (Hemiptera: Diaspididae). Annals of the Entomological Society of America 102: 1050-1061.

64. Weeks AR, Velten R, Stouthamer R (2003) Incidence of a new sex-ratio-distorting endosymbiotic bacterium among arthropods. Proceedings of the Royal Society of London Series B-Biological Sciences 270: 1857-1865.

65. Zchori-Fein E, Perlman SJ (2004) Distribution of the bacterial symbiont *Cardinium* in arthropods. Molecular Ecology 13: 2009-2016.
