# Supplementary material for: A Veritable Menagerie of Heritable Bacteria from Ants, Butterflies, and Beyond: Broad Molecular Surveys and a Systematic Review
Source: PLoS One. 2012 Dec 20;7(12):e51027. doi: 10.1371/journal.pone.0051027 (PMC3527441; doi:10.1371/journal.pone.0051027)
Supplement: Table S1 — PCR conditions used in this study. See separate file. (DOCX) [file pone.0051027.s007.docx]

| Assay | Primers | 25 mM Mg++ (μl per 10 μl reaction) | Annealing Temperature (°C) | Extension Time (seconds) |
| --- | --- | --- | --- | --- |
| *Arsenophonus* | Ars2F & 1513R | 1.0 | 55 | 120 |
| *Arsenophonus* | Ars23S-1 & Ars23S-2 | 1.0 | 55 | 120 |
| *Cardinium* | Card154F & Card1237R | 0.6 | 56 | 105 |
| *Hamiltonella* | T1279F & 35R | 0.6 | 56 | 90 |
| *Spiroplasma*^‡^ | 63F & TKSSp | 0.4 | 55 | 60 |
| *Spiroplasma*^#^ | 16STF1 & 16STR1 | 0.4 | 65-48.5 | 45 |
| *Spiroplasma*^†^ | cute493F & 1513R | 0.2 | 56 | 105 |
| *Wolbachia* | wsp81F & wsp691R | 1.0 | 55 | 60 |
| Enteric bacteria | F40 & R1060 | 1.0 | 57 | 120 |
| Eubacteria | 9Fa & 1513R | 1.0 | 56 | 120 |
| Host template quality | Ben & Jerry | 0.6 | 45 | 60 |

**Supplementary Table 1:** Variables for PCR reactions.

^‡^Full PCR cycling conditions were: 94°C for 240 seconds; 35 cycles of 95°C for 30 seconds, 55°C for 30 seconds, 72C for 60 seconds.

^#^Full PCR cycling conditions were: 94°C for 180 seconds; 15 cycles of 94°C for 30 seconds, 65-48.5°C for 45 seconds (start at 65°C and drop 1.1°C each cycle), and 72°C for 45 seconds; 20 cycles of 94°C for 30 seconds, 48°C for 45 seconds and 72°C for 45 seconds; and finally one cycle of 72°C for 5 minutes.

^†^Assay more broadly screens for Entomoplasmatales bacteria.
