# Supplementary material for: A Veritable Menagerie of Heritable Bacteria from Ants, Butterflies, and Beyond: Broad Molecular Surveys and a Systematic Review
Source: PLoS One. 2012 Dec 20;7(12):e51027. doi: 10.1371/journal.pone.0051027 (PMC3527441; doi:10.1371/journal.pone.0051027)
Supplement: Table S2 — PCR and sequencing primer information. See separate file. (DOCX) [file pone.0051027.s008.docx]

| **Primer name** | **Primer sequence (5’ 🡪 3’)** | **Primer utility** | **Gene (position within 16S)**^†^ | **Primer publication** |
| --- | --- | --- | --- | --- |
| Card211F | 5’ GGCGGTGTAAAATGAGCG 3’ | Diagnostic primer used in screening for *Cardinium* | 16S rRNA (211) | This study |
| Card1310R | 5’ TCAATCCGAACTGAGACAT 3’ | Diagnostic primer used in screening for *Cardinium* | 16S rRNA (1310) | This study |
| T1279F | 5’ CGAGGGAAAGCGGAACTCAG 3’ | Diagnostic primer used in screening for *Hamiltonella* | 16S rRNA (1267) | Russell et al. 2003 |
| 35R | 5’ CCTTCATCGCCTCTGACTGC 3’ | General primer paired with T1279F for *Hamiltonella* screening | 23S rRNA | Russell et al. 2003 |
| Ars2F | 5’ CTTCGGATGAACCCATAIGAG 3’ | Diagnostic primer used in screening for *Arsenophonus* | 16S rRNA (222) | This study |
| Ars 23S-1 | 5’ CGTTTGATGAATTCATAGTCAAA 3’ | Diagnostic primer used in screening for *Arsenophonus* | 23S rRNA | [73] |
| Ars 23S-2 | 5’ GGTCCTCCAGTTAGTGTTACCCAAC 3’ | Diagnostic primer used in screening for *Arsenophonus* | 23S rRNA | [73] |
| 16STF1 | 5’ GGTCTTCGGATTGTAAAGGTCTG 3’ | Diagnostic primer used in screening for *Spiroplasma* | 16S rRNA (416) | Haselkorn et al. 2009 |
| 16STR1 | 5’ GGTGTGTACAAGACCCGAGAA 3’ | Diagnostic primer used in screening for *Spiroplasma* | 16S rRNA (1380) | Haselkorn et al. 2009 |
| TKSSsp | 5’ TAGCCGTGGCTTTCTGGTAA 3’ | Diagnostic primer used in screening for *Spiroplasma* | 16S rRNA (509) | Mateos et al. 2006 |
| 63F | GCCTAATACATGCAAGTCGAAC | Diagnostic primer used in screening for *Spiroplasma* | 16S rRNA (59) | Fukatsu & Nikoh 2000 |
| cute493F | 5’ AGAAAGCCACGGCIAACTAT 3’ | Diagnostic primer used to amplify Entomoplasmatales as part of *Spiroplasma* screening | 16S rRNA (493) | Funaro et al. 2011 |
| wsp81F | 5’ -TGGTCCAATAAGTGATGAAGAAAC 3’ | Diagnostic *Wolbachia* PCR, sequencing | *wsp* | Zhou et al. 1998 |
| wsp691R | 5’ AAAAATTAAACGCTACTCCA 3’ | Diagnostic *Wolbacia* PCR, sequencing | *wsp* | Zhou et al. 1998 |
| Bloch382F | 5’ AACCCTGATGCAGCTATACCGT 3’ (note that several *Blochmannia* species end in “C” meaning that this is not an ideal diagnostic primer) | Diagnostic primer for *Blochmannia* screening | 16S rRNA (382) | This study |
| Bloch1233R | 5’ CACGTTTGTAGCCCTACTCA 3’ | Diagnostic primer for *Blochmannia* screening | 16S rRNA (1233) | This study |
| F40 | 5’ GCGGCAAGCCTAACACAT 3’ | General PCR screening for enteric bacteria | 16S rRNA (38) | Novakova and Hypsa 2007 |
| R1060 | 5’ CTTAACCCAACATTTCTCAACACGAG 3’ | General PCR screening for enteric bacteria | 16S rRNA (1094) | Novakova and Hypsa 2007 |
| 9Fa | 5’ GAGTTTGATCITIGCTCAG 3’ | Universal 16S rRNA PCR | 16S rRNA (9) | Russell et al. 2009b |
| 1513R | 5’ TACIGITACCTTGTTACGACTT 3’ | Universal 16S rRNA PCR; Diagnostic PCR when paired with diagnostic forward primers | 16S rRNA (1513) | Russell et al. 2009b |
| 559R | 5’ GTATTACCGCGGCTGCTGGCACG 3’ | Sequencing | 16S rRNA (536) | Russell et al. 2009b |
| 786F - i | 5’ GATTAGATACCCIGGTAG 3’ | Sequencing | 16S rRNA (786) | Funaro et al. 2011 |
| 1072R | 5’ CGAGCTGACGACAICCATGC 3’ | Sequencing | 16S rRNA (1072) | Russell et al. 2009b |
| 1406R | 5’ GACGGGCGGTGTGTRCA 3’ | Sequencing | (1407) | Funaro et al. 2011 |
| M13F | 5’ GTAAAACGACGGCCAG 3’ | Amplification of cloned products | pCR2.1 plasmid region flanking cloning site |  |
| M13R | 5’ CAGGAAACAGCTATGAC 3’ | Amplification of cloned products | pCR2.1 plasmid region flanking cloning site |  |
| Ben | 5’ GCTACTACATAATAKGTATCATG 3’ | PCR assay for on template quality | coI gene from insect mtDNA | Simon et al. 1994 |
| Jerry | 5’ CAACATTTATTTTGATTTTTTGG 3’ | PCR assay for on template quality | coI gene from insect mtDNA | Simon et a. 1994 |

**Supplementary Table 2:** PCR primers utilized for symbiont screening of *Arsenophonus*, *Cardinium*, *Hamiltonella*, *Spiroplasma*, and *Wolbachia*; for DNA sequencing; for universal PCR; and for amplification of cloned products.

^†^Position of 5’ primer end with respect to location within *E. coli* 16S rRNA gene (GenBank Accession # J01695) presented in parentheses, where relevant.
